# Diabetes medications and cancer risk associations: a systematic review and meta-analysis of evidence over the past 10 years

**DOI:** 10.1038/s41598-023-38431-z

**Published:** 2023-07-22

**Authors:** Yixian Chen, Fidela Mushashi, Surim Son, Parveen Bhatti, Trevor Dummer, Rachel A. Murphy

**Affiliations:** 1 School of Population and Public Health, University of British Columbia, Vancouver, British Columbia Canada; 2 BC Cancer, Vancouver, British Columbia Canada; 3 Department of Epidemiology and Biostatistics, Schulich School of Medicine and Dentistry, Western University, London, Ontario Canada; 4 Cancer Control Research, BC Cancer, Vancouver, British Columbia Canada

**Keywords:** Drug safety, Breast cancer, Cancer epidemiology, Cancer prevention, Lung cancer, Cancer, Metabolic disorders, Oncology, Cancer

## Abstract

Diabetes medications may modify the risk of certain cancers. We systematically searched MEDLINE, Embase, Web of Science, and Cochrane CENTRAL from 2011 to March 2021 for studies evaluating associations between diabetes medications and the risk of breast, lung, colorectal, prostate, liver, and pancreatic cancers. A total of 92 studies (3 randomized controlled trials, 64 cohort studies, and 25 case–control studies) were identified in the systematic review, involving 171 million participants. Inverse relationships with colorectal (n = 18; RR = 0.85; 95% CI = 0.78–0.92) and liver cancers (n = 10; RR = 0.55; 95% CI = 0.46–0.66) were observed in biguanide users. Thiazolidinediones were associated with lower risks of breast (n = 6; RR = 0.87; 95% CI = 0.80–0.95), lung (n = 6; RR = 0.77; 95% CI = 0.61–0.96) and liver (n = 8; RR = 0.83; 95% CI = 0.72–0.95) cancers. Insulins were negatively associated with breast (n = 15; RR = 0.90; 95% CI = 0.82–0.98) and prostate cancer risks (n = 7; RR = 0.74; 95% CI = 0.56–0.98). Positive associations were found between insulin secretagogues and pancreatic cancer (n = 5; RR = 1.26; 95% CI = 1.01–1.57), and between insulins and liver (n = 7; RR = 1.74; 95% CI = 1.08–2.80) and pancreatic cancers (n = 8; RR = 2.41; 95% CI = 1.08–5.36). Overall, biguanide and thiazolidinedione use carried no risk, or potentially lower risk of some cancers, while insulin secretagogue and insulin use were associated with increased pancreatic cancer risk.

## Introduction

The prevalence of type II diabetes, herein referred to as diabetes, continues to grow and, globally, will affect 7.7% (439 million) of adults aged 20 to 79 years old by 2030^1^. Epidemiological evidence suggests that diabetes is associated with a higher risk for many cancers, from 20 to 30% elevated breast or colorectal cancer to 97% elevated risk of intrahepatic cholangiocarcinoma or endometrial cancer^2,3^. These associations likely reflect shared etiology. For instance, tobacco use, physical inactivity, overweight, and poor diet are closely linked to cancer risk and progression^4^ and risk of diabetes^5–7^. Several biological mechanisms have been proposed to explain why diabetes may be associated with cancer, including inflammation, hyperglycemia, and hyperinsulinemia^8,9^. Furthermore, medications for diabetes may modify the association. Multiple epidemiologic studies have evaluated the association of diabetes medications with the risk of site-specific cancers in patients with diabetes^10,11^.

Previous systematic reviews either synthesized the effect of a single medication class on multiple cancer sites^12–14^ or the effects of multiple medication classes on overall cancer^15^. No systematic reviews have synthesized multiple associations among different diabetes medication classes and cancer sites in patients with diabetes, which limits our capacity to analyze evidence in the context of various mechanisms involved with diabetes treatment and underlying causes of cancers. To this end, we conducted a systematic review and meta-analyses of the latest randomized controlled trials (RCTs) and observational studies (cohort and case–control studies) published in the last 10 years that evaluated the effect of diabetes medications, namely biguanides, incretin-based medicines, alpha-glucosidase inhibitors (AGIs), insulin secretagogues, thiazolidinediones, and insulins, on the risk of developing any of study cancers, specifically breast, lung, colorectal, prostate, liver, or pancreatic cancers.

## Results

### Literature search

**Figure 1:**
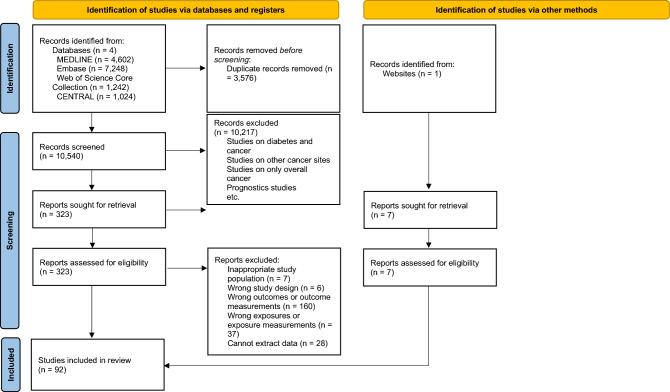
Flow diagram for selection of studies included in the systematic review and meta-analysis.

The literature search and study selection process are summarized in Fig. 1. Of the 10,540 citations uniquely identified from databases, 323 were eligible for full-text review. After review, 92 studies were selected for inclusion. A detailed description of these studies can be found in Supplementary file 1. A total of 226 separate associations between various diabetes medication classes and cancer sites were evaluated in the 92 studies (Table 1). Sodium-glucose cotransporter 2 inhibitors were included in the meta-analysis but will not be discussed in this review as no studies on cancer sites of interest were identified.

**Table 1:** Characteristics of the 92 studies selected for inclusion in systematic review and meta-analysis of diabetes medications and cancer associations.

	Overall	Study design			Risk of bias			Population region		
		RCT	Cohort	Case–control	Low	Moderate	High	Asian	Western	Multiple continents
Cancer site										
(1) Breast cancer	35	1	31	3	21	13	1	7	28	0
(2) Lung cancer	27	1	22	4	25	1	1	13	14	0
(3) Colorectal cancer	57	1	32	24	40	16	1	27	30	0
(4) Prostate cancer	31	1	19	11	27	3	1	6	24	1
(5) Liver cancer	34	1	20	13	27	6	1	17	17	0
(6) Pancreatic cancer	42	3	27	12	31	8	3	15	24	3
Medication class										
(1) Biguanides	64	0	44	20	45	19	0	21	42	1
(2) Incretin-based medicines	25	2	20	3	20	3	2	9	13	3
(3) Alpha-glucosidase inhibitors	6	0	4	2	6	0	0	5	1	0
(4) Insulin secretagogues	30	0	15	15	23	7	0	9	21	0
(5) Thiazolidinediones	46	6	26	14	36	4	6	20	26	0
(6) Insulins	55	0	42	13	41	14	0	21	34	0

### Study characteristics

All three RCTs were categorized as having a high risk of bias due to either lack of allocation concealment or potential selective reporting (Table 1 and supplementary references in Supplementary file 1). Among the 64 cohort studies, nine and 55 were categorized as moderate and low risk of bias, respectively. For the 55 retrospective cohort studies, observation time ranged from two to 25 years and 43 studies had data spanning more than five years. The follow-up period in nine prospective cohort studies varied from two to 14 years, and six studies followed participants for more than five years. Of the 25 case–control studies, nine and 16 were categorized as moderate and low risk of bias, respectively. Five of the case–control studies were conducted in hospital settings.

Overall, colorectal (n = 57) and pancreatic cancers (n = 42) were the most commonly evaluated outcomes across included studies (Table 1). A cohort design was used for the majority of the breast (89%) and lung cancer studies (81%). Studies of lung and prostate cancer were generally of the highest quality, with only 7% of lung and 13% of prostate cancer studies characterized as susceptible to moderate or high risk of bias. Regarding lung, colorectal, and liver cancers, approximately equivalent numbers of the studies were conducted in populations from Asian and Western regions. The majority of studies on breast, prostate, and pancreatic cancer were conducted in populations from Western regions.

Of the studies that reported specific diabetes medication classes, the majority studied biguanides and insulins, while only six studies evaluated the effect of AGIs on cancer risk. Most studies (67%) were of a cohort design. The proportions of studies with a low risk of bias were similar across diabetes medication classes. The majority of studies of biguanides and insulin secretagogues involved populations from Western regions, while the majority of studies of AGIs were conducted in populations from Asian regions.

### Meta-analysis

Individual associations among medication classes and cancer sites and subgroup analysis results are summarized in Tables 2, 3, 4, 5, 6 and 7. Forest plots for pooled analyses are shown in Supplementary file 2. Overall, a number of inverse associations were found. Biguanide use was significantly associated with lower risks of colorectal (n = 18; RR = 0.85; 95% CI = 0.78–0.92; Table 4) and liver cancers (n = 10; RR = 0.55; 95% CI = 0.46–0.66; Table 6). Use of thiazolidinediones was associated with lower risks of breast (n = 6; RR = 0.87, 95% CI = 0.80–0.95; Table 2), lung (n = 6; RR = 0.77; 95% CI = 0.61–0.96; Table 3) and liver cancers (n = 8; RR = 0.83; 95% CI = 0.72–0.95; Table 6). Lower risks of breast (n = 15; RR = 0.90; 95% CI = 0.82–0.98; Table 2) and prostate cancers (n = 7; RR = 0.74; 95% CI = 0.56–0.98; Table 5) were found among insulin users compared with nonusers. On the other hand, elevated risk of pancreatic cancer was observed with insulin secretagogue use (n = 5; RR = 1.26; 95% CI = 1.01–1.57; Table 7), and elevated risks of liver (n = 7; RR = 1.74; 95% CI = 1.08–2.80; Table 6) and pancreatic cancers (n = 8; RR = 2.41; 95% CI = 1.08–5.36; Table 7) were observed with insulin use.

**Table 2:** Diabetes medication classes and breast cancer risk.

		RR 95% CI	P value for effect	P value for Egger's test	I^2^ (%)	P value for difference between subgroups
Biguanides						
All studies (n = 8)		0.74 (0.47–1.17)	0.20	0.96	99.08	–
Study design	Cohort (n = 6)	0.69 (0.39–1.22)	0.21	–	99.01	0.37
	Case–control (n = 2)	0.92 (0.72–1.18)	0.51	–	77.07	
Risk of bias	Low risk (n = 4)	0.74 (0.36–1.51)	0.40	–	99.40	0.95
	Moderate risk (n = 4)	0.75 (0.54–1.06)	0.11	–	87.65	
Study population	Asian (n = 1)	0.38 (0.36–0.39)	< 0.001	–	–	< 0.001
	Western (n = 7)	0.84 (0.69–1.03)	0.09	–	89.12	
Incretin-based medicines						
All studies (n = 2)		0.78 (0.61–1.00)	0.05	0.46	44.34	–
Study design	Cohort (n = 2)	0.78 (0.61–1.00)	0.05	–	44.34	–
Risk of bias	Low risk (n = 1)	0.70 (0.56–0.87)	< 0.001	–	–	0.18
	Moderate risk (n = 1)	0.90 (0.67–1.22)	0.50	–	–	
Study population	Asian (n = 1)	0.70 (0.56–0.87)	< 0.001	–	–	0.18
	Western (n = 1)	0.90 (0.67–1.22)	0.50	–	–	
Insulin secretagogues						
All studies (n = 4)		1.04 (0.90–1.20)	0.60	0.97	9.80	–
Study design	Cohort (n = 4)	1.04 (0.90–1.20)	0.60	–	9.80	–
Risk of bias	Low risk (n = 3)	1.11 (0.96–1.30)	0.17	–	< 0.001	0.07
	Moderate risk (n = 1)	0.83 (0.63–1.10)	0.19	–	–	
Study population	Western (n = 4)	1.04 (0.90–1.20)	0.60	–	9.80	–
Thiazolidinediones						
All studies (n = 6)		0.87 (0.80–0.95)	< 0.001	0.75	< 0.001	–
Study design	RCT (n = 1)	0.52 (0.26–1.04)	0.07	–	–	0.14
	Cohort (n = 5)	0.88 (0.81–0.96)	< 0.001	–	< 0.001	
Risk of bias	Low risk (n = 4)	0.88 (0.80–0.96)	0.01	–	< 0.001	0.33
	Moderate risk (n = 1)	0.91 (0.68–1.20)	0.50	–	–	
	High risk (n = 1)	0.52 (0.26–1.04)	0.07	–	–	
Study population	Asian (n = 2)	0.87 (0.79–0.97)	0.01	–	< 0.001	0.93
	Western (n = 4)	0.87 (0.73–1.03)	0.10	–	< 0.001	
Insulins						
All studies (n = 15)		0.90 (0.82–0.98)	0.02	0.07	72.10	–
Study design	Cohort (n = 14)	0.87 (0.79–0.96)	< 0.001	–	67.70	< 0.001
	Case–control (n = 1)	1.15 (1.02–1.29)	0.03	–	–	
Risk of bias	Low risk (n = 9)	0.86 (0.76–0.96)	0.01	–	78.63	0.19
	Moderate risk (n = 6)	0.98 (0.83–1.14)	0.75	–	46.38	
Study population	Asian (n = 3)	0.79 (0.50–1.25)	0.31	–	41.44	0.61
	Western (n = 12)	0.89 (0.80–0.99)	0.04	–	76.47

**Table 3:** Diabetes medication classes and lung cancer risk.

		RR 95% CI	P value for effect	P value for Egger's test	I^2^ (%)	P value for difference between subgroups
Biguanides						
All studies (n = 9)		0.83 (0.68–1.02)	0.07	0.84	91.88	–
Study design	Cohort (n = 7)	0.78 (0.61–1.00)	0.05	–	91.82	0.06
	Case–control (n = 2)	1.03 (0.90–1.19)	0.65	–	53.26	
Risk of bias	Low risk (n = 8)	0.80 (0.65–0.98)	0.03	–	92.57	0.03
	Moderate risk (n = 1)	1.44 (0.89–2.33)	0.13	–	–	
Study population	Asian (n = 4)	0.70 (0.47–1.03)	0.07	–	94.90	0.18
	Western (n = 5)	0.94 (0.78–1.14)	0.54	–	84.29	
Incretin-based medicines						
All studies (n = 2)		0.81 (0.50–1.31)	0.39	0.04	78.27	–
Study design	Cohort (n = 2)	0.81 (0.50–1.31)	0.39	–	78.27	–
Risk of bias	Low risk (n = 2)	0.81 (0.50–1.31)	0.39	–	78.27	–
Study population	Western (n = 2)	0.81 (0.50–1.31)	0.39	–	78.27	–
Alpha-glucosidase inhibitors						
All studies (n = 1)		0.56 (0.35–0.90)	0.02	–	–	–
Study design	Cohort (n = 1)	0.56 (0.35–0.90)	0.02	–	–	–
Risk of bias	Low risk (n = 1)	0.56 (0.35–0.90)	0.02	–	–	–
Study population	Asian (n = 1)	0.56 (0.35–0.90)	0.02	–	–	–
Insulin secretagogues						
All studies (n = 3)		0.99 (0.90–1.10)	0.90	0.94	< 0.001	–
Study design	Cohort (n = 2)	0.96 (0.74–1.23)	0.73	–	< 0.001	0.75
	Case–control (n = 1)	1.00 (0.89–1.12)	0.99	–	–	
Risk of bias	Low risk (n = 3)	0.99 (0.90–1.10)	0.90	0.94	< 0.001	–
Study population	Asian (n = 1)	1.17 (0.69–1.97)	0.56	–	–	0.53
	Western (n = 2)	0.99 (0.89–1.10)	0.81	–	< 0.001	
Thiazolidinediones						
All studies (n = 6)		0.77 (0.61–0.96)	0.02	0.87	54.31	–
Study design	RCT (n = 1)	1.27 (0.65–2.49)	0.49	–	–	0.13
	Cohort (n = 5)	0.73 (0.58–0.93)	0.01	–	56.66	
Risk of bias	Low risk (n = 5)	0.73 (0.58–0.93)	0.01	–	56.66	0.13
	High risk (n = 1)	1.27 (0.65–2.49)	0.49	–	–	
Study population	Asian (n = 3)	0.72 (0.47–1.11)	0.14	–	67.96	0.83
	Western (n = 3)	0.77 (0.56–1.05)	0.09	–	36.81	
Insulins						
All studies (n = 6)		1.04 (0.79–1.37)	0.79	0.36	94.94	–
Study design	Cohort (n = 5)	0.97 (0.68–1.39)	0.87	–	95.94	0.13
	Case–control (n = 1)	1.31 (1.14–1.51)	< 0.001	–	–	
Risk of bias	Low risk (n = 6)	1.04 (0.79–1.37)	0.79	–	94.94	–
Study population	Asian (n = 4)	1.11 (0.85–1.45)	0.46	–	59.00	0.76
	Western (n = 2)	1.01 (0.60–1.68)	0.97	–	97.08

**Table 4:** Diabetes medication classes and colorectal cancer risk.

		RR 95% CI	P value for effect	P value for Egger's test	I^2^ (%)	P value for difference between subgroups
Biguanides						
All studies (n = 18)		0.85 (0.78–0.92)	< 0.001	0.11	97.33	–
Study design	Cohort (n = 11)	0.80 (0.71–0.92)	< 0.001	–	98.28	0.15
	Case–control (n = 7)	0.91 (0.82–1.00)	0.05	–	88.63	
Risk of bias	Low risk (n = 12)	0.86 (0.78–0.95)	< 0.001	–	98.12	0.51
	Moderate risk (n = 6)	0.81 (0.70–0.94)	< 0.001	–	78.74	
Study population	Asian (n = 7)	0.76 (0.62–0.94)	0.01	–	98.49	0.13
	Western (n = 11)	0.90 (0.84–0.96)	< 0.001	–	80.05	
Incretin-based medicines						
All studies (n = 5)		1.03 (0.99–1.06)	0.11	0.34	< 0.001	–
Study design	Cohort (n = 4)	1.01 (0.97–1.06)	0.58	–	< 0.001	0.34
	Case–control (n = 1)	1.04 (1.00–1.09)	0.07	–	–	
Risk of bias	Low risk (n = 4)	1.03 (1.00–1.06)	0.10	–	< 0.001	0.36
	Moderate risk (n = 1)	0.06 (0.01–27.59)	0.36	–	–	
Study population	Asian (n = 2)	1.03 (0.99–1.06)	0.11	–	< 0.001	0.78
	Western (n = 3)	0.99 (0.76–1.28)	0.93	–	19.23	
Alpha-glucosidase inhibitors						
All studies (n = 2)		1.13 (0.97–1.31)	0.11	0.43	93.78	–
Study design	Cohort (n = 1)	1.21 (1.17–1.26)	< 0.001	–	–	< 0.001
	Case–control (n = 1)	1.04 (0.98–1.11)	0.18	–	–	
Risk of bias	Low risk (n = 2)	1.13 (0.97–1.31)	0.11	–	93.78	–
Study population	Asian (n = 2)	1.13 (0.97–1.31)	0.11	–	93.78	–
Insulin secretagogues						
All studies (n = 9)		1.07 (0.95–1.21)	0.25	0.33	94.82	–
Study design	Cohort (n = 4)	1.21 (1.04–1.40)	0.01	–	85.06	0.04
	Case–control (n = 5)	1.03 (0.97–1.08)	0.38	–	40.29	
Risk of bias	Low risk (n = 6)	1.10 (0.97–1.26)	0.15	–	95.75	0.22
	Moderate risk (n = 3)	1.00 (0.94–1.07)	0.96	–	< 0.001	
Study population	Asian (n = 4)	1.13 (0.97–1.32)	0.13	–	97.10	0.18
	Western (n = 5)	1.01 (0.95–1.07)	0.83	–	< 0.001	
Thiazolidinediones						
All studies (n = 11)		1.00 (0.96–1.03)	0.86	0.19	55.38	–
Study design	RCT (n = 1)	0.74 (0.43–1.27)	0.27	–	–	0.50
	Cohort (n = 4)	0.94 (0.76–1.17)	0.59	–	63.45	
	Case–control (n = 6)	0.99 (0.96–1.03)	0.72	–	52.91	
Risk of bias	Low risk (n = 8)	1.01 (0.96–1.06)	0.74	–	60.70	0.37
	Moderate risk (n = 2)	0.97 (0.91–1.04)	0.38	–	62.65	
	High risk (n = 1)	0.74 (0.43–1.27)	0.27	–	–	
Study population	Asian (n = 7)	1.01 (0.98–1.04)	0.54	–	46.96	0.08
	Western (n = 4)	0.87 (0.73–1.03)	0.10	–	37.73	
Insulins						
All studies (n = 12)		1.04 (0.94–1.16)	0.42	0.39	95.19	–
Study design	Cohort (n = 8)	1.03 (0.86–1.22)	0.77	–	96.81	0.75
	Case–control (n = 4)	1.06 (0.97–1.15)	0.19	–	59.90	
Risk of bias	Low risk (n = 8)	1.03 (0.91–1.17)	0.63	–	96.58	0.54
	Moderate risk (n = 4)	1.15 (0.83–1.61)	0.41	–	87.21	
Study population	Asian (n = 5)	1.22 (1.01–1.46)	0.04	–	94.15	0.09
	Western (n = 7)	0.96 (0.79–1.18)	0.72	–	95.68

**Table 5:** Diabetes medication classes and prostate cancer risk.

		RR 95% CI	P value for effect	P value for Egger's test	I^2^ (%)	P value for difference between subgroups
Biguanides						
All studies (n = 11)		0.87 (0.62–1.21)	0.40	0.94	99.60	–
Study design	Cohort (n = 7)	0.75 (0.45–1.25)	0.27	–	99.75	0.14
	Case–control (n = 4)	1.10 (1.05–1.16)	< 0.001	–	< 0.001	
Risk of bias	Low risk (n = 9)	0.88 (0.56–1.40)	0.59	–	99.50	0.98
	Moderate risk (n = 2)	0.87 (0.59–1.30)	0.50	–	59.24	
Study population	Asian (n = 1)	0.33 (0.31–0.34)	< 0.001	–	–	< 0.001
	Western (n = 9)	0.99 (0.93–1.06)	0.76	–	74.95	
	Multiple continents (n = 1)	1.20 (0.84–1.73)	0.31	–	–	
Incretin-based medicines						
All studies (n = 1)		0.59 (0.47–0.73)	< 0.001	–	–	–
Study design	Cohort (n = 1)	0.59 (0.47–0.73)	< 0.001	–	–	–
Risk of bias	Low risk (n = 1)	0.59 (0.47–0.73)	< 0.001	–	–	–
Study population	Asian (n = 1)	0.59 (0.47–0.73)	< 0.001	–	–	–
Insulin secretagogues						
All studies (n = 4)		1.01 (0.96–1.06)	0.71	0.29	< 0.001	–
Study design	Cohort (n = 2)	1.03 (0.91–1.16)	0.68	–	< 0.001	0.90
	Case–control (n = 2)	1.02 (0.94–1.10)	0.68	–	33.56	
Risk of bias	Low risk (n = 4)	1.01 (0.96–1.06)	0.71	–	< 0.001	–
Study population	Western (n = 4)	1.01 (0.96–1.06)	0.71	–	< 0.001	–
Thiazolidinediones						
All studies (n = 8)		0.98 (0.86–1.11)	0.77	0.66	31.66	–
Study design	RCT (n = 1)	1.01 (0.56–1.81)	0.98	–	–	0.96
	Cohort (n = 4)	1.00 (0.79–1.27)	1.00	–	49.24	
	Case–control (n = 3)	0.96 (0.78–1.18)	0.70	–	53.78	
Risk of bias	Low risk (n = 7)	0.98 (0.85–1.12)	0.76	–	41.40	0.92
	High risk (n = 1)	1.01 (0.56–1.81)	0.98	–	–	
Study population	Asian (n = 1)	0.54 (0.19–1.53)	0.25	–	–	0.26
	Western (n = 7)	0.99 (0.87–1.12)	0.87	–	32.94	
Insulins						
All studies (n = 7)		0.74 (0.56–0.98)	0.04	0.10	92.88	–
Study design	Cohort (n = 5)	0.75 (0.50–1.14)	0.18	–	94.94	0.74
	Case–control (n = 2)	0.81 (0.66–1.01)	0.06	–	34.76	
Risk of bias	Low risk (n = 6)	0.80 (0.59–1.09)	0.16	–	93.16	0.02
	Moderate risk (n = 1)	0.51 (0.40–0.64)	< 0.001	–	–	
Study population	Asian (n = 3)	1.16 (0.65–2.06)	0.61	–	63.76	0.06
	Western (n = 4)	0.61 (0.42–0.87)	0.01	–	92.90

**Table 6:** Diabetes medication classes and liver cancer risk.

		RR 95% CI	P value for effect	P value for Egger's test	I^2^ (%)	P value for difference between subgroups
Biguanides						
All studies (n = 10)		0.55 (0.46–0.66)	< 0.001	0.58	87.45	–
Study design	Cohort (n = 8)	0.56 (0.45–0.68)	< 0.001	–	89.57	0.96
	Case–control (n = 2)	0.55 (0.38–0.80)	< 0.001	–	52.76	
Risk of bias	Low risk (n = 7)	0.52 (0.43–0.64)	< 0.001	–	91.49	0.15
	Moderate risk (n = 3)	0.69 (0.51–0.93)	0.02	–	< 0.001	
Study population	Asian (n = 5)	0.54 (0.43–0.68)	< 0.001	–	93.62	0.73
	Western (n = 5)	0.57 (0.44–0.75)	< 0.001	–	40.40	
Incretin-based medicines						
All studies (n = 2)		0.95 (0.67–1.36)	0.79	0.47	< 0.001	–
Study design	Cohort (n = 1)	0.93 (0.65–1.33)	0.69	–	–	0.46
	Case–control (n = 1)	1.90 (0.29–12.38)	0.50	–	–	
Risk of bias	Low risk (n = 2)	0.95 (0.67–1.36)	0.79	–	< 0.001	–
Study population	Asian (n = 1)	0.93 (0.65–1.33)	0.69	–	–	0.46
	Western (n = 1)	1.90 (0.29–12.38)	0.50	–	–	
Alpha-glucosidase inhibitors						
All studies (n = 2)		1.34 (0.36–4.98)	0.67	0.33	86.18	–
Study design	Cohort (n = 1)	0.73 (0.52–1.03)	0.07	–	–	0.01
	Case–control (n = 1)	2.82 (1.12–7.09)	0.03	–	–	
Risk of bias	Low risk (n = 2)	1.34 (0.36–4.98)	0.67	–	86.18	–
Study population	Asian (n = 1)	0.73 (0.52–1.03)	0.07	–	–	0.01
	Western (n = 1)	2.82 (1.12–7.09)	0.03	–	–	
Insulin secretagogues						
All studies (n = 5)		1.30 (0.89–1.89)	0.17	0.96	82.22	–
Study design	Cohort (n = 1)	0.78 (0.57–1.08)	0.14	–	–	0.01
	Case–control (n = 4)	1.49 (1.06–2.09)	0.02	–	71.21	
Risk of bias	Low risk (n = 3)	1.34 (0.74–2.42)	0.33	–	89.70	0.86
	Moderate risk (n = 2)	1.25 (0.76–2.05)	0.38	–	67.61	
Study population	Asian (n = 2)	1.11 (0.56–2.21)	0.77	–	87.61	0.53
	Western (n = 3)	1.45 (0.90–2.33)	0.13	–	80.62	
Thiazolidinediones						
All studies (n = 8)		0.83 (0.72–0.95)	0.01	0.02	63.67	–
Study design	RCT (n = 1)	0.80 (0.22–2.98)	0.74	–	–	0.19
	Cohort (n = 4)	0.64 (0.44–0.95)	0.03	–	57.50	
	Case–control (n = 3)	0.92 (0.86–1.00)	0.04	–	45.15	
Risk of bias	Low risk (n = 7)	0.82 (0.72–0.95)	0.01	–	68.79	0.97
	High risk (n = 1)	0.80 (0.22–2.98)	0.74	–	–	
Study population	Asian (n = 5)	0.83 (0.72–0.96)	0.01	–	76.66	0.56
	Western (n = 3)	0.70 (0.41–1.21)	0.20	–	< 0.001	
Insulins						
All studies (n = 7)		1.74 (1.08–2.80)	0.02	0.19	92.90	–
Study design	Cohort (n = 5)	1.46 (0.94–2.29)	0.10	–	86.54	0.48
	Case–control (n = 2)	2.35 (0.69–8.00)	0.17	–	96.95	
Risk of bias	Low risk (n = 6)	1.86 (1.06–3.29)	0.03	–	93.94	0.23
	Moderate risk (n = 1)	1.26 (0.93–1.71)	0.14	–	–	
Study population	Asian (n = 3)	1.03 (0.68–1.57)	0.89	–	71.08	0.02
	Western (n = 4)	2.48 (1.31–4.70)	0.01	–	93.45

**Table 7:** Diabetes medication classes and pancreatic cancer risk.

		RR 95% CI	P value for effect	P value for Egger's test	I^2^ (%)	P value for difference between subgroups
Biguanides						
All studies (n = 8)		1.08 (0.77–1.52)	0.65	0.53	96.10	–
Study design	Cohort (n = 5)	1.01 (0.62–1.64)	0.98	–	97.18	0.43
	Case–control (n = 3)	1.25 (1.03–1.50)	0.02	–	43.74	
Risk of bias	Low risk (n = 5)	1.05 (0.69–1.59)	0.83	–	97.74	0.97
	Moderate risk (n = 3)	1.04 (0.78–1.39)	0.80	–	< 0.001	
Study population	Asian (n = 3)	0.97 (0.51–1.83)	0.92	–	98.56	0.62
	Western (n = 5)	1.15 (0.91–1.45)	0.23	–	62.28	
Incretin-based medicines						
All studies (n = 13)		0.73 (0.47–1.11)	0.14	0.56	96.69	–
Study design	RCT (n = 2)	0.76 (0.20–2.95)	0.69	–	28.67	0.53
	Cohort (n = 10)	0.70 (0.42–1.19)	0.19	–	97.33	
	Case–control (n = 1)	0.96 (0.81–1.12)	0.58	–	–	
Risk of bias	Low risk (n = 10)	0.68 (0.42–1.09)	0.11	–	97.10	0.02
	Moderate risk (n = 1)	1.41 (1.13–1.75)	< 0.001	–	–	
	High risk (n = 2)	0.76 (0.20–2.95)	0.69	–	28.67	
Study population	Asian (n = 4)	0.84 (0.41–1.69)	0.62	–	96.77	0.67
	Western (n = 6)	0.63 (0.24–1.65)	0.34	–	97.79	
	Multiple continents (n = 3)	0.95 (0.81–1.12)	0.55	–	< 0.001	
Alpha-glucosidase inhibitors						
All studies (n = 1)		1.15 (0.99–1.34)	0.06	–	–	–
Study design	Cohort (n = 1)	1.15 (0.99–1.34)	0.06	–	–	–
Risk of bias	Low risk (n = 1)	1.15 (0.99–1.34)	0.06	–	–	–
Study population	Asian (n = 1)	1.15 (0.99–1.34)	0.06	–	–	–
Insulin secretagogues						
All studies (n = 5)		1.26 (1.01–1.57)	0.04	0.79	86.08	–
Study design	Cohort (n = 2)	1.09 (1.01–1.17)	0.02	–	< 0.001	0.04
	Case–control (n = 3)	1.46 (1.11–1.91)	0.01	–	74.71	
Risk of bias	Low risk (n = 4)	1.31 (1.01–1.69)	0.04	–	89.37	0.31
	Moderate risk (n = 1)	1.05 (0.77–1.45)	0.74	–	–	
Study population	Asian (n = 2)	1.09 (1.01–1.17)	0.02	–	< 0.001	0.04
	Western (n = 3)	1.46 (1.11–1.91)	0.01	–	74.71	
Thiazolidinediones						
All studies (n = 7)		0.73 (0.47–1.14)	0.17	0.66	86.70	–
Study design	RCT (n = 1)	0.31 (0.12–0.85)	0.02	–	–	0.01
	Cohort (n = 4)	0.59 (0.39–0.89)	0.01	–	69.18	
	Case–control (n = 2)	1.26 (0.79–2.02)	0.33	–	67.60	
Risk of bias	Low risk (n = 5)	0.77 (0.44–1.34)	0.35	–	89.99	0.12
	Moderate risk (n = 1)	0.98 (0.64–1.48)	0.91	–	–	
	High risk (n = 1)	0.31 (0.12–0.85)	0.02	–	–	
Study population	Asian (n = 2)	0.80 (0.37–1.75)	0.58	–	76.96	0.75
	Western (n = 5)	0.68 (0.35–1.34)	0.27	–	88.92	
Insulins						
All studies (n = 8)		2.41 (1.08–5.36)	0.03	0.61	99.01	–
Study design	Cohort (n = 5)	2.92 (0.98–8.70)	0.06	–	99.25	0.43
	Case–control (n = 3)	1.77 (0.95–3.30)	0.07	–	92.94	
Risk of bias	Low risk (n = 6)	2.06 (1.55–2.73)	< 0.001	–	77.20	0.62
	Moderate risk (n = 2)	3.60 (0.40–32.23)	0.25	–	99.38	
Study population	Asian (n = 3)	2.16 (1.52–3.09)	< 0.001	–	< 0.001	0.76
	Western (n = 5)	2.56 (0.92–7.14)	0.07	–	99.42	

Effects were largely consistent across studies with different designs, quality levels, and population regions, though there are some notable differences. Subgroup analyses revealed that the positive association between insulin use and liver cancer was restricted to studies conducted in populations from Western regions (n = 4; RR = 2.48, 95% CI = 1.31–4.70; Table 6). The use of insulin secretagogues was significantly associated with higher colorectal cancer risk in cohort studies (n = 4; RR = 1.21; 95% CI = 1.04–1.40; Table 4) but not in case–control studies. Biguanide use was associated with a higher risk of pancreatic cancer in case–control studies but not in cohort studies (n = 3; RR = 1.25; 95% CI = 1.03–1.50; Table 7). Thiazolidinedione use was associated with a lower risk of pancreatic cancer in cohort studies (n = 4; RR = 0.59; 95% CI = 0.39–0.89; Table 7), whereas, in case–control studies, a non-significant increased risk of pancreatic cancer was observed (n = 2; RR = 1.26, 95% CI = 0.79–2.02).

## Discussion

This comprehensive meta-analysis examined associations among the most commonly prescribed diabetes medication classes and the risk of cancer in patients with diabetes. The meta-analysis included 92 primary studies which involved 171 million participants, of which 1.3 million were diagnosed with cancer, from across 18 countries or regions. Evidence of increased and decreased risks of cancer was found. For example, biguanide use was associated with a moderately decreased risk of colorectal cancer and a considerably lower risk of liver cancer. Thiazolidinedione use was found to moderately reduce the risks of breast, lung, and liver cancers. Conversely, the use of insulin secretagogues was associated with a moderately elevated risk of pancreatic cancer. Opposing associations with different cancer sites within a medication class were also evident; insulin use was associated with a higher risk of liver and pancreatic cancers but a lower risk of breast and prostate cancers. In addition to varied cancer-specific biologic mechanisms, these differences may be attributed to variations in research instruments and the composition of study populations (e.g., age differences) across the included studies.

Several biological mechanisms have been speculated to contribute to cancer modifying effects of diabetes medications. The protective effects of biguanides on colorectal cancer may be attributed to their ability to regulate upstream and downstream molecular mechanisms involved in cellular metabolism and energy homeostasis, cell cycle arrest, oxidative stress, inflammation, and apoptosis^16^. The potential protective association of biguanides with liver cancer might be further attributed to decreased lipogenic enzyme gene expression and suppressed lipogenesis^17^. By stimulating endogenous insulin production, insulin secretagogues increase insulin-like growth factor-1 levels which may promote pancreatic cancer development by interfering with cell metabolism and stimulating cell proliferation^18^. Thiazolidinediones have been shown to suppress breast cancer cell proliferation, stimulate apoptosis, and impede tumor angiogenesis with PPAR-γ ligands^19^. Thiazolidinediones-induced PPAR-γ activation has also been shown to hinder lung tumor progression through G0/G1 cell cycle arrest^20^. The inverse association observed between thiazolidinediones and liver cancer may be attributed to p27^Kip1^ protein accumulation which is associated with reduced liver cell growth^21^.

A protective effect of insulin was observed on breast cancer in the review, but the etiology is unclear. The lack of adjustment for mammography screening suggested detection bias might be a concern. Our finding of lower prostate cancer risk in men with diabetes using insulins is similar to a previous study. Men who were on insulins were more likely to have severe diabetes which might be linked to lower prostate-specific antigen levels and reduced risk of prostate cancer^22^. The cancer-promoting effect of insulins on the liver might be partly explained by portal circulation, which exposes the liver to high levels of insulin in people who have insulin-resistant diabetes^23^. In addition, insulin use has growth-promoting and mitogenic effects on pancreatic cancer cells, which may lead to pancreatic cancer development or acceleration^24^. However, the higher risk of pancreatic cancer might also be attributable to severe diabetes conditions rather than insulin use.

Although Noto et al. ^25^ found that individuals with diabetes had an overall increased cancer risk, our study highlights the impact of specific diabetes medications on site-specific cancer risk. In alignment with the systematic review and meta-analysis by Noto et al. ^26^, our findings underscore the potential protective effect of biguanides, such as metformin, in reducing the risk of colorectal and liver cancers. Similarly, the protective effect of metformin in our study echoes DeCensi et al. ^27^, implying a robust consensus across studies regarding this association. Our findings that insulin and insulin secretagogue use can be linked to an elevated risk of certain cancers align with the results from Karlstad et al. ^28^ and Singh et al. ^29^. These consistent findings across studies highlight the potentially harmful effects of these medication classes on cancer risk, meriting careful clinical consideration. In addition to confirming results from previous systematic reviews, our review broadens the understanding by incorporating more recent studies, diverse geographical regions, and differing study designs, thus enhancing the comprehensiveness and applicability of the findings.

Our meta-analysis reveals associations between diabetes medication use and cancer risk that were generally consistent across diverse study designs, quality levels, and population regions. However, regional differences were discovered, such as the stronger association between insulin use and liver cancer risk in populations from the Western regions. The inconsistent association between insulin secretagogues and colorectal cancer and biguanides and pancreatic cancer, which varied by study design, underscores the need to interpret results cautiously. Our findings indicate that geographical factors and study design might influence observed associations, necessitating additional research to enhance our understanding.

This review represents the latest examination of associations among all currently prescribed classes of diabetes medications and the risk of cancer across multiple sites. A myriad of new diabetes medications has become available over the past two decades^30^. Our approach, considering different classes of medications separately across individual cancer sites, is an important advance over previous literature reviews^12–15^ that have grouped all diabetes medications and/or cancer sites together. The large sample size of included studies augmented the precision of summary effect estimates, and the inclusion of large numbers of individuals from Asian and Western regions enhances the generalizability of our findings.

Our review has some limitations. Evidence of heterogeneity in summary risk estimates was observed for biguanides and insulins across all cancer sites, but subgroup analyses did not reveal clear sources of this heterogeneity. It is possible that heterogeneity reflects differences in study population demographics such as varying proportions of males/females and differences in age or unmeasured confounding within studies, for example, few studies adjusted for lifestyle factors like nutritional status that may confound associations. It also appeared that study design impacted results. For instance, the use of insulin secretagogues showed a more prominent effect on liver and pancreatic cancers in case–control studies than in cohort studies. When the majority of studies were case–control studies, differences observed in results between cohort and case–control studies might be attributed to recall bias. Effects of sodium-glucose cotransporter 2 inhibitors on cancer risk and AGIs on breast and prostate cancer could not be explored due to the absence of data. In general, few studies of cancer risk in association with AGIs and incretin-based medicines were identified. Differences in the standard of care for diabetes across jurisdictions and the use of combination drug therapies may have impacted results. Because the severity of diabetes is typically a driver of specific medication use, the possibility of confounding by indication cannot be ruled out in the individual studies that were included in this review. We did not evaluate dose–response relationships by incorporating the duration of diabetes medication use because it is beyond the scope of this review.

In conclusion, various diabetes medications might be linked to the risk of study cancers; however, the strengths of associations showed variation to some extent across studies with different designs, quality levels, and population regions. On the other hand, the preventive effect of biguanides against colorectal and liver cancers was consistent across different studies. A potential cancer-promoting effect of insulins on the liver was only found in populations from Western regions, whereas the increased risk associated with insulin on the pancreas was only found in populations from Asian regions. Although further studies are required, these findings suggest that it may be important to weigh the potential harms of insulin among patients with diabetes who are at high risk of liver or pancreatic cancers due to family history or other risk factors. The choice of medication for patients with diabetes may incorporate individuals’ susceptibility to individual cancers in conjunction with established clinical considerations. Future studies may further consider the duration of medication use and dose–response relationships to consolidate current knowledge in observed associations with study cancers.

## Methods

The search adhered to the guidelines provided by the Preferred Reporting Items for Systematic Reviews and Meta-analyses (PRISMA)^31^ statement. The completed PRISMA checklist can be found in Supplementary file 3. This systematic review protocol was registered with the International Prospective Register of Systematic Reviews (PROSPERO) in March 2021 (registration number: CRD42021239348).

### Study design

We included original human experimental and observational studies that evaluated the associations between diabetes medications and six site-specific cancers: breast, lung, colorectal, prostate, liver, and pancreatic cancers. The first four cancers are the most prevalent cancers worldwide ^32^, and thus it is critical to understand possible risk factors. As a result of the high prevalence, we also expected to identify a larger body of evidence for breast, lung, colorectal and prostate cancer from which to synthesize findings. Although cancers of the liver and pancreas are less common, they have high fatality rates. Moreover, liver and pancreatic cancers were included in this review due to biological plausibility of diabetes medication and liver and pancreatic cancer, reflecting the roles of the liver and pancreas in blood glucose regulation and diabetes. Studies using a cross-sectional design were excluded as they could not provide clear evidence of temporality. In this study, we reviewed the results of RCTs, cohort, and case–control studies. Reviews, commentaries, opinion pieces, letters, and case reports were also excluded.

### Participants

The study population for breast cancer was restricted to female biological sex due to the rareness of breast cancer in males. People with prevalent cancer or a previous history of study cancers were excluded. Studies focusing on pediatric or adolescent populations were also excluded. No other restrictions were placed on the study population.

### Exposures

In RCTs, diabetes medications were randomly assigned. For observational studies, the determination of the exposure to diabetes medications was made on the basis of medical records, pharmacy prescription records, or insurance claims databases. Self-reported exposure was also included, recognizing its inherent limitations. Studies were excluded if they did not provide explicit methodologies for establishing exposure to diabetes medication.

### Comparators

Studies without an appropriate comparator group were excluded. In addition to people receiving no treatment or placebo, for each diabetes medication of interest, people using one or more other diabetes medications were considered to be eligible as controls. Control groups were from population-based and hospital settings in observational studies and in RCTs, respectively.

### Outcomes

Cancer incidence or mortality was determined through a national record system, cancer registry or death certificates. Eligible measures of the effect included odds ratio (OR), relative risk (RR), and hazard ratio (HR). The absence of these specific effect measures led to exclusion.

### Publication characteristics

This systematic review focuses on research published in the last ten years. Searches were limited to publications dating from January 2011 onwards. The timeframe was chosen to reflect advances in pharmaceutical treatment of diabetes; the introduction of new medications e.g., GLP-1 receptor agonists and SGT-2 inhibitors with distinct mechanisms of action from existing diabetes medication classes. The focus on recent literature is thus more likely to capture evidence on diabetes medications currently prescribed and have greater clinical relevance. No language restrictions were imposed. After the titles/abstracts screening, the full text of selected studies in languages other than English was translated using Google Translate.

### Search strategies

With the assistance of an institutional librarian, we systematically searched MEDLINE (Ovid), Embase (Ovid), Web of Science Core Collection, and Cochrane CENTRAL (Ovid). Search terms included controlled vocabulary and keywords related to various diabetes medications and cancer at multiple sites. Details of the search strategy for each database are provided in Supplementary file 4.

### Study selection

All titles and abstracts were imported to Covidence (https://www.covidence.org). The study selection comprised two stages. Firstly, all titles and abstracts were screened by reviewers (Y.C., F.M., and S.S.) for potentially eligible papers according to the above-mentioned inclusion and exclusion criteria. Studies that were excluded by more than one reviewer were excluded from this review. Secondly, full texts of all relevant studies were retrieved, and two reviewers (Y.C., F.M., or S.S.) independently screened each study for eligibility. Reasons for exclusion were documented at the full-text screening stage.

### Data extraction

Two reviewers, Y.C. and F.M. or Y.C. and S.S., extracted data from each included study using Covidence independently. Discrepancies in extracted data were resolved through discussion or consensus with a third reviewer.

### Quality assessment

The methodological quality of RCTs and observational studies was scrutinized independently by two reviewers and collated by the lead reviewer using Cochrane Collaboration’s tool for assessing the risk of bias^33^ and the Newcastle–Ottawa scale (NOS)^34^. If randomization, allocation concealment, and blinding were uncertain in an RCT, it was categorized as a high-risk study^15^. Three domains assessed in observational studies were the selection of study groups, the comparability of groups, and the ascertainment of exposure or outcome of interest. A score of less than 4 or more than 6 represented a high or low risk of bias, respectively^15^.

### Data synthesis

We performed meta-analyses to obtain the pooled relative risk (RR) using Stata software version 17.0 (Stata Corp., College Station, TX, USA). First, the findings were summarized in a narrative form, with textual description and tabulation to compare potential differences among studies. We employed a DerSimonian-Laird random-effects model^35^ to pool the data from two or more studies evaluating the same class of diabetes medication and the same site of cancer. Cochran’s Q was used to test the statistical heterogeneity of the included studies, with a significance level of 0.1^36^. The magnitude of heterogeneity was measured using the I^2^ statistic^37^, with < 25% and > 75% corresponding to low and high heterogeneity, respectively. Analyses were performed to quantify individual associations among different medication classes and cancer sites. We performed subgroup analyses (study design, quality levels, and population regions) to determine possible sources of between-study heterogeneity.

## Supplementary Information

Supplementary Information 1.

Supplementary Information 2.

Supplementary Information 3.

Supplementary Information 4.

## Data Availability

Data are available upon request from Y.C. (ychen153@student.ubc.ca).

## Abbreviations

AGIs: Alpha-glucosidase inhibitors
Cochrane CENTRAL: Cochrane Central Register of Controlled Trials
EMBASE: Excerpta Medica DataBase
HR: Hazard ratio
MEDLINE: Medical Literature Analysis and Retrieval System Online
NOS: Newcastle–Ottawa scale
OR: Odds ratio
PRISMA: Preferred Reporting Items for Systematic Reviews and Meta-Analyses
PROSPERO: Prospective Register of Systematic Reviews
RCT: Randomized controlled trial
RR: Relative risk
95% CI: 95% Confidence interval
