# Supplementary material for: Diabetes medications and cancer risk associations: a systematic review and meta-analysis of evidence over the past 10 years
Source: Sci Rep. 2023 Jul 22;13:11844. doi: 10.1038/s41598-023-38431-z (PMC10363143; doi:10.1038/s41598-023-38431-z)
Supplement: Supplementary file 1 — Supplementary Information 1. [file 41598_2023_38431_MOESM1_ESM.docx]

**Diabetes medications and cancer risk associations: a systematic review and meta-analysis of evidence over the past 10 years**

**Author Details**

* Yixian Chen: School of Population and Public Health, University of British Columbia, Vancouver, Canada. ychen153@student.ubc.ca

Fidela Mushashi: BC Cancer, Vancouver, Canada. fidela.mushashi@bccancer.bc.ca

Surim Son: Department of Epidemiology and Biostatistics, Schulich School of Medicine & Dentistry, Western University, London, Ontario, Canada. sson8@uwo.ca

Parveen Bhatti: Cancer Control Research, BC Cancer and School of Population and Public Health, University of British Columbia, Vancouver, Canada. pbhatti@bccrc.ca

Trevor Dummer: School of Population and Public Health, University of British Columbia, Vancouver, Canada. trevor.dummer@ubc.ca

Rachel A. Murphy: Cancer Control Research, BC Cancer and School of Population and Public Health, University of British Columbia, Vancouver, Canada. rachel.murphy@ubc.ca

**Corresponding Author**

Yixian Chen: School of Population and Public Health, University of British Columbia, Vancouver, Canada. ychen153@student.ubc.ca

Mailing address: Rm 167-2206 East Mall, Vancouver, BC V6T 1Z3, Canada

**SUPPLEMENTARY FILE 1**

**Table 1. Characteristics of included studies assessing cancer risk with diabetes medications**

| Study | Location  /setting | Time period; mean/median follow-up (years) | Age range, mean/median and standard deviation/IQR (years) | Percent male (%) | Exposure/treatment categories | Outcome | Matched/adjusted/randomized factors | Risk of bias | NOS score |
| --- | --- | --- | --- | --- | --- | --- | --- | --- | --- |
| 3 Randomized controlled trials (RCTs) |  |  |  |  |  |  |  |  |  |
| Hernandez 2018 [1] | 28 countries; hospital-based | 2015 to 2018; median 1.6 | 40+; 64.1 (8.7) in albiglutide group; 64.2 (8.7) in placebo group | 69.4 | Class 2, albiglutide vs no use of albiglutide | Pancreatic cancer | Randomized by age, sex, race/ethnicity, geographic region, current smoking, medical history, body mass index, glycated haemoglobin, estimated glomerular filtration rate (EGFR), duration of diabetes, history of microvascular disease, cardiovascular medications, and glucose-lowering medications | High | NA |
| Jones 2015 [2] | 364 centers in 25 countries in Europe and Australasia; hospital-based | 2008 to 2012; NA | 40 to 75; 58.4 (8.3) in rosiglitazone group; 58.5 (8.3) in metformin/sulfonylurea group | 51.4 in rosiglitazone group; 51.7 in metformin/sulfonylurea group | Class 5, rosiglitazone combined with sulfonylurea or metformin vs metformin and sulfonylurea | Breast, lung, colorectal, prostate, liver, and pancreatic cancers | Randomized by age, gender, ethnicity, body weight, body mass index, menopause status, duration of diabetes, HbA1c | High | NA |
| Bethel 2017 [3] | 38 countries; hospital-based | NA; median 2.9 | 50+; 82% were <75 | 71.1 | Class 2, sitagliptin vs no use of sitagliptin | Pancreatic cancer | NA | High | NA |
| 64 Cohort studies |  |  |  |  |  |  |  |  |  |
| Soffer 2015 [4] | United States; population-based | 1998 to 2009; median 6 | 18+; 55.5 (16.1) | NA | Class 1, metformin vs no use of metformin | Breast cancer | Adjusted for age of index diabetes diagnosis, race/ethnicity, ERT status, statin use, Charlson comorbidity index, and outpatient utilization | Low | 9 |
| Tsai 2014 [5] | Taiwan; population-based | 1997 to 2007; NA | 15+; 54.4 (14.2) | 53.2 | Class 1, metformin vs never use of metformin | Lung cancer | Adjusted for age, gender, and modified Charlson Comorbidity Index score | Low | 7 |
| Habel 2013 [6] | United States; population-based | 2001 to 2009; median 3.3 | ~60% were >50 | 53.1 in glargine users; 49.7 in neutral protamine hagedorn (NPH) insulin users | Class 6, glargine vs NPH insulin use only | Breast, colorectal, and prostate cancers | Adjusted for Kaiser Permanente region, calendar year, age, metformin use, and use of short-acting insulin | Moderate | 6 |
| Knapen 2016 [7] | United Kingdom; population-based | 2007 to 2012; mean 4.1 | 18+; 58.1 (11.8) in incretins exposed group; 62.4 (14.9) in the exposed group of other noninsulin antidiabetic medications | 56.3 | Class 2, incretins vs use of other noninsulin antidiabetic medications | Pancreatic cancer | Matched on sex, year of birth (within 5 years) and practice;  Adjusted for alcoholism, alcohol use, body mass index, smoking, neuropathy, and retinopathy | Low | 8 |
| Funch 2018 [8] | United States; population-based | 2010 to 2014; NA | 18+; median 52.0 (IQR: 44.0, 58.0) in liraglutide exposed group; 51.0 (IQR: 39, 59) in liraglutide unexposed group | NA | Class 2, liraglutide vs all comparator drugs except exenatide (to remove any GLP-1 RA effect) | Breast cancer | Propensity score matched on demography, smoking, alcohol, comorbidity, concomitant medications, and intensity of health care utilization | Moderate | 6 |
| Chang 2011 [9] | Taiwan; population-based | 2004 to 2007; mean 2.0 | 18+; 60.7 (12.8) in insulin glargine users; 62.1 (13.4) in intermediate/long-acting human insulin (HI) users | 49.5 in insulin glargine initiators; 47.7 in intermediate/long-acting HI users | Class 6, insulin glargine vs intermediate/long-acting HI | Breast, lung, colorectal, prostate, liver, and pancreatic cancers | Propensity score adjusted for age category, sex (if shared sites), initiation year, diabetes-related late complications, major comorbidities, Charlson Index (not include diabetes), other diabetes medications, statins, aspirin, resource utilization, and physician characteristics, time-varying medication use, and dosage of studied insulin | Low | 7 |
| Suissa 2011 [10] | United Kingdom; population-based | 2002 to 2009; NA | 40+; 60.9 (13.1) in glargine group; 61.2 (12.2) in other insulin group | NA | Class 6, insulin glargine vs other insulin treatment (including human long-acting insulins, human biphasic insulins, short-acting analogue, and animal insulins) | Breast cancer | Adjusted for age categories, age, excessive alcohol use, smoking status, obesity (body mass index>30 kg/m^2^), HbA1c, diabetes duration, duration of insulin use, oophorectomy, history of cancer, use of hormone replacement therapy, sulfonylureas, thiazolidinediones, metformin and statins | Low | 8 |
| Lai 2012a [11] | Taiwan; population-based | 1995 to 2008; NA | 20+; 56.4 (13.4) | 55.7 | Class 1, metformin vs no use of metformin;  Class 3, alpha-glucosidase inhibitors vs no use of alpha-glucosidase inhibitors;  Class 4, sulfonylureas vs no use of sulfonylureas;  Class 5, thiazolidinediones vs no use of thiazolidinediones;  Class 6, insulin vs no use of insulin | Lung cancer | Adjusted for sex, age, pulmonary tuberculosis, chronic obstructive pulmonary disease, and propensity score (quintile) | Low | 7 |
| Funch 2014 [12] | United States; population-based | 2010 to 2012; NA | 18+; median 53.0 | 45.8 in liraglutide users; 50.5 in other users | Class 2, liraglutide vs pooled comparator drugs (excluding exenatide and DPP-4 inhibitors) | Pancreatic cancer | Adjusted for age, gender, healthcare utilization, and Diabetes Complications and Severity Index | Low | 7 |
| Tseng 2014a [13] | Taiwan; population-based | 1996 to 2009; NA | NA; ~54.8% of human insulin ever users and ~49.6% never users were >60, respectively | 100.0 | Class 6, human insulin vs no use of human insulin | Prostate cancer | Adjusted for age, diabetes duration, benign prostatic hyperplasia, major comorbidities, diabetes-related complications, other cancer before baseline, other diabetes medications, commonly used medications, aspirin, nonsteroidal anti-inflammatory drug, anticoagulants, and 5-alpha reductase inhibitor | Low | 8 |
| Vallarino 2013 [14] | United States; population-based | 2000 to 2010; mean 2.2 for pioglitazone and 1.9 for insulin patients | 45+; 58.1 (8.7) in pioglitazone cohort and 59.7 (10.3) in insulin cohort | 59.6 in pioglitazone cohort; 53.0 in insulin cohort | Class 5, pioglitazone vs insulin | Breast, lung, colorectal, prostate, and pancreatic cancers | Adjusted for demographics (age, tobacco use), use of medications (other antidiabetic drugs, commonly used cardiovascular medications, aspirins, NSAIDs, anticoagulants, antiplatelets, etc.) and medical history (obesity, hyperlipidemia, myocardial infarction, coronary heart disease, coronary revascularization stroke, congestive heart failure, hypertension, arrhythmias, cancer, bone fracture, renal impairment, gout) | Low | 7 |
| Schlesinger 2013 [15] | 10 European countries; population-based | 1992 to 2000; mean 8.5 | 25 to 70; 57.7 (7.9) | 47.5 | Class 6, insulin vs no use of insulin | Liver cancer | Stratified by sex, center, and age at recruitment;  Adjusted for age, education, smoking status, baseline alcohol consumption, body mass index, and waist-to-height ratio | Low | 9 |
| Gu 2013 [16] | Shanghai, China; population-based | 2002 to 2010; NA | 30+; 62.2 (18.0) in insulin users; 61.0 (17.0) in non-insulin users | 51.5 in insulin users; 54.4 in non-insulin users | Class 6, insulin vs not use of insulin | Breast, lung, colorectal, prostate, liver, and pancreatic cancers | Adjusted for age, smoking status, diabetes duration, macrovascular, HbA1c and concomitant oral glucose-lowering agents (including metformin, sulfonylureas, and acarbose) | Low | 7 |
| Kao 2013 [17] | Taiwan; population-based | 2001 to 2009; NA | 20+; 56.5 (13.2) | 55.1 | Class 5, thiazolidinediones vs no use of thiazolidinediones | Breast, lung, colorectal, prostate, liver, and pancreatic cancers | Matched on birth year, sex, and calendar year of diabetes diagnosis | Low | 7 |
| Fagot 2013 [18] | France; population-based | 2007 to 2009; median 2.67, 2.75, and 2.83 in patients exposed to insulin glargine, insulin detemir, and BHI, respectively. | 40+; overall population (both men and women) 63.2; 10.10 | NA | Class 6, insulin glargine or insulin detemir or basal human insulin (BHI) vs no use of Insulin glargine, insulin detemir BHI | Breast cancer | Adjusted for use of other hypoglycemic agents and duration of diabetes | Moderate | 6 |
| Redaniel 2012 [19] | United Kingdom; population-based | 1987 to 2007; NA | 35+; ~51.5% were >60 | NA | Class 4, sulfonylurea vs no use of sulfonylurea;  Class 6, insulin vs no use of insulin | Breast cancer | Adjusted for age, period, region, body mass index, year of diagnosis, and weighted HbA1c | Low | 9 |
| Sakoda 2015 [20] | United States; population-based | 1997 to 2012; mean 9.0 | 40+; 59.3 (10.1) in metformin users; 65.0 (11.5) in non-users | 52.3 in metformin users and 55.3 in non-users | Class 1, metformin vs never use of metformin | Lung cancer | Stratified on age and adjusted for gender, race/ethnicity, birth year, diabetes duration, body mass index, alcohol use, Charlson comorbidity index, smoking history (status and pack-years), education, income level, creatinine level, HbA1c level, and use of other diabetes medications | Low | 8 |
| Calip 2016 [21] | United States; population-based | 1996 to 2011; median 6.7 | 40+; 61.6 (12.3) | NA | Class 1, metformin vs no use of metformin;  Class 4, sulfonylureas vs no use of sulfonylureas;  Class 6, all insulins vs no use of insulin | Breast cancer | Adjusted for other diabetes medications, age at cohort entry, study entry year, smoking status, menopausal status, Charlson score, statin use, and menopausal hormone therapy | Low | 8 |
| Wang 2016 [22] | United States; population-based | 2002 to 2012; mean 6.4 | 40-89; 70.2 (9.5), 65.1 (11.0), and 68.8 (10.0) in non-Hispanic White, African American, and Hispanic nonusers of metformin; 64.4 (9.3), 59.0 (9.5), and 63.1 (9.3) in non-Hispanic White, African American, and Hispanic users of metformin | 100.0 | Class 1, metformin vs no use of metformin | Prostate cancer | Adjusted for indicators of African American, Hispanics, metformin use, statin and finasteride use, two-way interactions of medication indicators, interactions between race/ethnicity with variables involving metformin use (i.e., indicator of metformin use, the product of the metformin use indicator and statin use indicator, and the product of metformin use indicator and finasteride use indicator), age, change in LDL, HbA1c, and body mass index, and the maximum prostate specific antigen level during the study period;  Inverse probability weights adjusted for baseline HbA1c, age, and Charlson comorbidity score | Low | 8 |
| Erdmann 2016 [23] | 19 European countries; population-based | 10 years; mean 7.8 | 35 to 75; 61.8 (7.7) | 66.1 | Class 5, pioglitazone vs no use of any diabetes medications | Breast, lung, colorectal, prostate, liver, and pancreatic cancers | NA | Low | 7 |
| Tseng 2016 [24] | Taiwan; population-based | 1999 to 2010; NA | 25+; 52.6 (11.5) in ever-users of sitagliptin; 56.4 (13.8) in never users | 53.8 in ever-users of sitagliptin; 52.9 in never-users of sitagliptin | Class 2, sitagliptin vs no use of sitagliptin | Pancreatic cancer | Propensity score adjusted for age, sex, major comorbidities, diabetes-related complications, acute pancreatitis, gallstone, commonly used medications, other diabetes medications, aspirin, and anticoagulants | Low | 8 |
| Tseng 2015 [25] | Taiwan; population-based | 1996 to 2009; NA | NA; 63.7 (12.7) in ever-users of human insulin; 62.5 (12.2) in never-users of human insulin | NA | Class 6, human insulin vs no use of human insulin | Breast cancer | Adjusted for age, diabetes duration, major comorbidities, diabetes-related complications, other cancer prior to baseline, other diabetes medications, commonly used medications, non-steroidal anti-inflammatory drugs, and estrogen | Low | 8 |
| Tuccori 2015 [26] | United Kingdom; population-based | 1988 to 2013; mean 5.3 | 40+; 64.0 (11.9) in users of glyburide; 64.0 (12.5) in users of other second-generation sulfonylureas | 66.4 in users of glyburide; 73.3 in users of other second-generation sulfonylureas | Class 4, glyburide vs use of other second-generation sulfonylureas | Breast, lung, colorectal, and prostate cancers | Adjusted for age, body mass index, smoking status, excessive alcohol use, HbA1c, duration of treated diabetes, comedications, use of other antidiabetic medications, ever use of oral contraceptives, hormone replacement therapy, and previous mammography screening | Low | 8 |
| Tseng 2014b [27] | Taiwan; population-based | 1998 to 2009; NA | 40+; 57.1 (10.6) in ever-users of metformin; 62.6 (12.4) in never-users of metformin; | 100.0 | Class 1, metformin vs no use of metformin | Prostate cancer | Propensity score adjusted for age, major comorbidities, diabetes-related complications, urinary tract disease, benign prostatic hyperplasia, other cancers, commonly used medications, other diabetes medications, aspirin, anticoagulants, non-steroidal anti-inflammatory drugs, and prostate specific antigen | Low | 8 |
| Gokhale 2014 [28] | United States; population-based | 2007 to 2011; median 10 months (IQR: 5, 18 months) | 65+; mean 75.0 | 35.0 | Class 2, dipeptidyl peptidase-4 inhibitors vs sulfonylureas or thiazolidinediones | Pancreatic cancer | Propensity score weighted for comorbidities and health care utilization | Low | 7 |
| Tsilidis 2014 [29] | United Kingdom; population-based | 1987 to 2010; median 5.1 | 35 to 90; 61.1 in users of metformin monotherapy; 65.3 in users of sulfonylurea monotherapy | 56.1 in users of metformin monotherapy; 57.9 in users of sulfonylurea monotherapy | Class 1, metformin monotherapy vs sulfonylurea monotherapy | Breast (postmenopausal breast cancer), lung, colorectal, prostate, liver, and pancreatic cancers | Adjusted for smoking status, body mass index, alcohol consumption, use of aspirin or NSAIDs, statins, diabetes duration, and year of first anti-diabetes prescription | Low | 8 |
| Tseng 2014c [30] | Taiwan; population-based | 1998 to 2009; NA | NA; 56.5 (12.3) in ever-users of metformin; 54.9 (16.7) in never-users of metformin | NA | Class 1, metformin vs no use of metformin | Breast cancer | Adjusted for age, major comorbidities, diabetes-related complications, other cancer, commonly used medications, other antidiabetic medications, aspirin, nonsteroidal anti-inflammatory drugs, and anticoagulants | Low | 7 |
| Vicentini 2018 [31] | Italy; population-based | 2009 to 2014; NA | 20 to 84; 66.1 (11.1) | 55.6 | Class 1, metformin vs untreated diabetes (diet only) | Breast, lung, colorectal, prostate, liver, and pancreatic cancers | Adjusted for age, citizenship, and time since diagnosis of diabetes | Moderate | 6 |
| Bradley 2018 [32] | United States; population-based | 1995 to 2012; mean 9.0 | 40+; 48.7% of ever users and 69.0% never users of metformin were >60, respectively | 53.95; 52.3 in ever users; 55.3 in never users | Class 1, metformin vs never use of metformin | Colorectal cancer | Adjusted for gender, race/ethnicity, birth year, diabetes duration, body mass index, alcohol use, smoking status, Charlson comorbidity index, education, income level, creatinine, HbA1c, history of lower endoscopy; first lower endoscopy after baseline (time-varying) and use of other types of diabetes medications (time-varying) | Low | 8 |
| Tseng 2017a [33] | Taiwan; population-based | 1999 to 2011; NA | 25 to 75; 61.4 (10.2) in ever users of metformin; 63.8 (10.4) in never users of metformin | 53.7 in ever-users of metformin; 57.2 in never-users of metformin | Class 1, metformin vs no use of metformin | Lung cancer | Inverse probability of treatment weighting using propensity score adjusted for age, sex, occupation, living region, major comorbidities, diabetes-related complications, other antidiabetic drugs, potential risk factors of cancer, medications that are commonly used in diabetes patients and may affect cancer risk | Low | 8 |
| Tseng 2017b [34] | Taiwan; population-based | 1995 to 2005; median 5.5 | 25 to 75; 62.0 (10.1) | 54.2 | Class 1, metformin vs no use of metformin | Colorectal cancer | Inverse probability of treatment weighting using propensity score adjusted for age, gender, occupation, region of residence, major comorbidities, diabetes-related complications, antidiabetic drugs, diagnosis possibly associated with cancer risk, medications commonly used for diabetes that may affect cancer risk, and entry date | Low | 9 |
| Abrahami 2018 [35] | United Kingdom; population-based | Up to 9.5 years; NA | NA; 64.0 (12.0) | 58.2 | Class 2,  GLP-1 analogues or DPP-4 inhibitors  vs use of other diabetes medication(s) | Colorectal cancer | Adjusted for  age, sex, year of cohort entry, body mass index, smoking, alcohol-related disorders (including, e.g., alcoholism, alcoholic cirrhosis of the liver, alcoholic hepatitis, and hepatic failure), hemoglobin A1c, duration of treated diabetes, previous cancer, presence of inflammatory bowel disease, Charlson comorbidity score, neuropathy, renal disease, retinopathy, peripheral arteriopathy, aspirin, statins, the number of unique antidiabetic drugs, and the total number of unique nondiabetic drugs in the year before cohort entry | Low | 9 |
| Boniol 2018 [36] | Belgium and Italy; population-based | NA; Belgium exposed mean 1.81; Belgium unexposed mean 2.83; Italy exposed mean 1.25; Italy unexposed mean 2.55 | 18+; >70% are 50+ | 49.5 | Class 2, GLP-1 RA (sexenatide and liraglutide) and the DPP-4 inhibitors (sitagliptin, vildagliptin, saxagliptin, alogliptin, and linagliptin) vs noninsulin antidiabetic drug;  Class 6, insulin vs no prescription of insulin | Pancreatic cancer | Stratified by age and sex and adjusted for use of insulin | Moderate | 6 |
| Tseng 2018a [37] | Tai; population-based | 1999 to 2005; NA | 45+; NA | NA | Class 5,  pioglitazone vs no use of pioglitazone | Lung cancer | Propensity score matching adjusted for age, sex, diabetes duration, living region, occupation and the following factors: hypertension, chronic obstructive pulmonary disease (COPD),tobacco abuse, stroke, nephropathy, ischemic heart disease, peripheral arterial disease, eye disease, obesity, dyslipidemia, insulin, sulfonylurea, metformin, meglitinide, acarbose, statin, fibrate, angiotensin converting enzyme inhibitor/angiotensin receptor blocker, calcium channel blocker, aspirin, ticlopidine, clopidogrel, and dipyridamole | Low | 7 |
| You 2020 [38] | South Korea; population-based | 2005 to 2014; NA | NA; 60.7 (15.4) in users of metformin; 60.9 (13.9) in non-users of metformin | 49.9 in users of metformin; 50.9 in non-users of metformin | Class 1, metformin vs no use of metformin | Colon, liver, and pancreatic cancers | Adjusted for age, sex, economic status, region of residency, and antidiabetic medications (sulfonylureas, thiazolidinediones, dipeptidyl peptidase 4 inhibitors, alpha-glucosidase inhibitors, and meglitinide) | Low | 8 |
| Rouette 2020 [39] | United Kingdom; population-based | 2007 to 2017; median 4.6 | 18+; 60.7 (13.6) | 57.1 | Class 2, dipeptidyl peptidase-4 inhibitors or glucagon-like peptide-1 receptor agonist vs other second- or third-line antidiabetic drugs (including initiating on combination therapy or switching to or adding-on a new antidiabetic drug class) | Lung cancer | Adjusted for age, sex, year of cohort entry, body mass index, HbA1c, duration of treated diabetes, macrovascular complications (stroke, myocardial infarction, peripheral vascular disease), microvascular complications (neuropathy, nephropathy and retinopathy), smoking status, alcohol-related disorders (including alcoholism, alcoholic cirrhosis of the liver, alcoholic hepatitis, hepatic failure), chronic obstructive pulmonary disease, use of statins, and total number of unique non-diabetes drugs | Low | 9 |
| Haring 2017 [40] | Finland; population-based | 1995 to 2009; NA | 55.0 to 67.0; median 59.0 | 100.0 | Class 1, metformin vs no use of metformin;  Class 4, sulfonylurea vs no use of sulfonylurea;  Class 5, thiazolidinedione vs no use of thiazolidinedione | Prostate cancer | Adjusted for age, trial arm (prostate specific antigen screening at 4-year intervals or Standard care), and use of antihypertensives, cholesterol-lowering drugs, 5-alpha-reductase inhibitors, alpha-blockers, non-steroidal anti-inflammatory drugs and aspirin | Low | 7 |
| Chen 2017 [41] | Canada; population-based | 1994 to 2012; median 8.3 in exposed group; median 10.4 in unexposed group | 50+; 65.3 (9.5) in exposed group; 62.7 (8.5) in unexposed group | 100.0 | Class 1, metformin vs no metformin | Prostate cancer | Adjusted for socioeconomic class, number of physician visits and use of other diabetes medications (sulfonylureas, acarbose, meglitinides, dipeptidyl peptidase IV inhibitors, glucagon-like peptide 1 receptor agonists, thiazolidinediones and insulin) | Moderate | 6 |
| Tseng 2017c [42] | Taiwan; population-based | 1999 to 2010; NA | 25+; 53.6 (11.3) in ever users of sitagliptin; 55.9 (13.3) in never users of sitagliptin | NA | Class 2, sitagliptin vs no use of sitagliptin | Breast cancer | Inverse probability of treatment weighting using propensity score adjusted for age, diabetes-related comorbidities, acute pancreatitis, commonly used medications, other diabetes medications, aspirin, anticoagulant medications, estrogen, and potential detection examinations | Low | 8 |
| Tseng 2017d [43] | Taiwan; population-based | 1996 to 2009; NA | Over 85% were aged >50 | NA | Class 5, rosiglitazone vs no use of rosiglitazone | Breast cancer | Adjusted for age, diabetes duration, hypertension, COPD, cerebrovascular disease, nephropathy, ischemic heart disease, peripheral arterial disease, eye disease, obesity, dyslipidemia, benign breast conditions, other cancer prior to baseline, sulfonylurea, metformin, acarbose, insulin, estrogen, potential detection examinations | Low | 8 |
| Bowker 2017 [44] | United States; population-based | 6 years; NA | 30+; 53.0 (9.2) | NA | Class 1, metformin vs no use of metformin;  Class 4, sulfonylurea vs use of other diabetes medication(s);  Class 5, thiazolidinedione vs no use of thiazolidinedione;  Class 6, insulin vs no use of insulin | Breast cancer | Adjusted for age at drug index | Moderate | 6 |
| Tseng 2017e [45] | Taiwan; population-based | 1997 to 2012; NA | 18+; 64.1 (12.2) in incretin cohort; 63.6 (12.2) in non-incretin cohort | 50.5 | Class 2, incretin-based medicines (including saxagliptin, vildagliptin, sitagliptin, exenatide, and linagliptin) vs no use of incretin-based medicines | Pancreatic cancer | Matched on age (±1 year), gender, Diabetes Complication Severity Index;  Adjusted for gallstone disease, alcohol-related disease, hypertriglyceridemia, pancreatic disease, obesity, and tobacco use | Low | 7 |
| Tseng 2017f [46] | Taiwan; population-based | 1999 to 2010; NA | 25+; 51.5 (11.5) in users; 54.2 (12.9) in non-users | 100.0 | Class 2, sitagliptin vs no use of sitagliptin | Prostate cancer | Inverse probability of treatment weighting using propensity score adjusted for age, diabetes duration, hypertension, COPD, stroke, diabetes-related comorbidities, acute pancreatitis, benign prostatic hyperplasia, commonly used medications, other diabetes medications, and prostate-specific antigen | Low | 8 |
| Tseng 2019 [47] | Taiwan; population-based | 1996 to 2009; NA | NA; ~61% of ever users of human insulin and 55% of never users were >60 | 46.1 in ever users of human insulin; 50.0 in never users of human insulin | Class 6, human insulin vs no use of human insulin | Lung cancer | Adjusted for age category, sex, diabetes duration, comorbidities, other cancer prior to baseline, other diabetic medications, other commonly used medications, number of outpatient visits per year | Low | 8 |
| Erkinantti 2020 [48] | Finland; population-based | 1996 to 2011; NA | 40+; over 50% were >70 | 52.3 | Class 1, metformin vs no use of metformin;  Class 6, insulin vs no use of insulin | Colon cancer | Adjusted for age, sex, and the duration of diabetes | Low | 7 |
| Lee 2019a [49] | South Korea; population-based | 2007 to 2013; mean 3.6 in DPP-4i users; 3.4 in nonusers | 40 to 79; 62.1; 9.2 | 57.8 | Class 2, dipeptidyl peptidase 4 inhibitors (including sitagliptin, vildagliptin, linagliptin, saxagliptin, and gemigliptin) vs no use of dipeptidyl peptidase 4 inhibitors | Pancreatic cancer | Adjusted for age, sex, body mass index, smoking status, alcohol intake, Charlson comorbidity index, residential region, use of antidiabetic drug (biguanides, sulfonylureas, TZDs, and insulin), histories of cholecystectomy, and gastrectomy | Low | 8 |
| Liang 2019 [50] | United States; population-based | 2005 to 2015; median 0.9 in exposed group; median 1.3 in unexposed group | NA; more than 60% were aged >50 | 50.0 | Class 2, exenatide vs no use of exenatide | Pancreatic cancer | Adjusted for unbalanced propensity score model variables that were identified from a separate database, including number of antidiabetic drugs, metformin, pharmacy cost, and number of unique drugs dispensed at baseline | Low | 7 |
| Chang 2018 [51] | Taiwan; population-based | 1996 to 2008; mean 7.2 | 15+; 54.4 (14.2) | 53.1 | Class 1, metformin vs no use of metformin | Colorectal cancer | Adjusted for age, gender, modified Charlson comorbidity index score (subtracting two diabetic conditions from the original Charlson Comorbidity Index score) | Low | 7 |
| Albai 2020 [52] | Romania; hospital-based | 2015 to 2018; NA | 41 to 88; 66.3 (10.0) | 43.4 | Class 1, metformin vs no use of metformin;  Class 2, incretins vs no incretins;  Class 4, sulfonylureas vs no sulfonylureas;  Class 6, insulin vs no insulin | Colorectal cancer | Adjusted for age, gender, body mass index, abdominal circumference, smoking, alcohol, diabetes mellitus duration, HbA1, triglycerides, total cholesterol, HDLc, LDLc, hypertension | Moderate | 5 |
| Yen 2020 [53] | Taiwan; population-based | 2000 to 2012; mean 5.0 in metformin users; 5.2 in nonusers | 40 to 100; 65.6 (12.5) in users of metformin; 61.8 (10.7) in non-users | 54.9 | Class 1, metformin vs no use of metformin | Lung cancer | Propensity score matching adjusted for gender, age category, Charlson Comorbidity Index, moderate and severe exacerbation of COPD, bacterial pneumonia, Diabetes Complications Severity Index score, antihypertensive drugs, other antidiabetic drugs, respiratory drugs, and other drugs | Low | 8 |
| Bradley 2020 [54] | United States; population-based | 2006 to 2017; median 2.9 in glargine users, median 2.5 in detemir users, and 2.3 in NPH users | 65+; ~50% were aged between 65 and 74 | NA | Class 6, glargine vs neutral protamine hagedorn insulin or detemir insulin | Breast cancer | Inverse probability of treatment weighting using propensity score adjusted for age, race, low-income status, zip code-level income, metropolitan statistical area, medication use, medical conditions, alcohol abuse, smoking, and health care utilization | Low | 7 |
| Chen 2020 [55] | Taiwan; population-based | 1998 to 2010; mean 6.8 | 20+; 57.6 (13.2) | 53.5 | Class 6, insulin vs no use of insulin | Colorectal cancer | Adjusted for age, sex, urbanization level, comorbidities, insulin usage and examinations | Low | 7 |
| Wang 2019 [56] | United States; population-based | 2001 to 2012; NA | 40 to 89; 65.4 (9.6) in users of metformin; 71.2 (10.2) in non-users | 100.0 | Class 1, metformin vs no use of metformin | Liver cancer | Adjusted for age, age-adjusted Charlson comorbidity score, statin use, beta-blocker use, baseline and temporal change of body mass index, low-density lipoprotein (LDL) and hemoglobin A1c (HbA1c), having had diagnosed for alcohol related mental health disorders, nonspecific abnormal liver functions during the study period, and patient's residential neighborhood SES | Low | 8 |
| Kincius 2020 [57] | Lithuania; population-based | 2000 to 2016; ~45% were followed up for >5 years | 40+; ~66% were > 60 | 100.0 | Class 1, metformin vs no use of metformin | Prostate cancer | Adjusted for age | Low | 7 |
| Yen 2021 [58] | Taiwan; population-based | 2007 to 2012; NA | 18 to 80; 35 and 41% of DPP-4 inhibitor users and nonusers were > 65 years old | 67.0 | Class 2, dipeptidyl Peptidase-4 (DPP-4) inhibitor vs no use of DPP-4 inhibitor | Liver cancer | Adjusted for sex, age, age of diabetes mellitus diagnosis, Charlson comorbidity index, Diabetes Complications Severity Index score, angiotensin-converting enzyme inhibitors/angiotensin receptor blockers, beta-blockers, calcium-channel blockers, diuretics, other antihypertensive, metformin, sulfonylureas, meglitinides, alpha-glucosidase inhibitors, thiazolidinedione, insulin, statins, aspirin, hepatitis B virus and hepatitis C virus infection | Low | 7 |
| Tseng 2018b [59] | Taiwan; population-based | 1999 to 2005; median 5.3 | 25 to 74; 59.6 (12.2) in ever users of metformin; 64.5 (13.5) never users | 47.2 in ever users; 45.6 in never users | Class 1, metformin vs no use of metformin | Liver cancer | Inverse probability of treatment weighting using propensity score adjusted for age, sex, occupation, residential area, major comorbidities, diabetes-related complications, antidiabetic drugs, potential risk factors for cancer, medications that are commonly used among diabetes patients or that may affect cancer risk | Low | 7 |
| Lee 2018 [60] | South Korea; population-based | 2009 to 2015; median 4.9 | 30+; 57.2 (12.5) | 55.6 | Class 1, metformin vs no use of metformin;  Class 2,  dipeptidyl peptidase-4 inhibitor vs no use of dipeptidyl peptidase-4 inhibitor;  Class 3, alpha-glucosidase inhibitor vs no use of alpha-glucosidase inhibitor;  Class 4, sulfonylurea or meglitinide vs no sulfonylurea and no meglitinide;  Class 5,  thiazolidinedione vs no use of thiazolidinedione;  Class 6, insulin vs no use of insulin | Pancreatic cancer | Adjusted for age, sex, chronic pancreatitis, acute pancreatitis, hepatitis B, hepatitis C, biliary disease, alcoholism, non-alcoholic fatty liver disease, income of lowest quartile, place of residence, and the number of different exposed anti-diabetic medications | Low | 8 |
| Lee 2019c [61] | South Korea; population-based | 2009 to 2015; median 5.4 | 20+; 68.7% of colorectal cancer cases and 45.5% of controls were >60 | 66.7 in colorectal cancer cases; 59.9 in controls | Class 1, metformin vs no use of metformin;  Class 2, dipeptidyl peptidase-4 inhibitor vs no use of dipeptidyl peptidase-4 inhibitor;  Class 3, acarbose vs no use of acarbose;  Class 4, sulfonylurea or meglitinide vs no use of sulfonylurea and meglitinide;  Class 5, thiazolidinedione vs no use of thiazolidinedione;  Class 6 insulin vs use of insulin | Colorectal cancer | Adjusted for age, sex, smoking, alcohol consumption, regular exercise, yearly income, residency, body mass index, waist circumference, hypertension, dyslipidemia, complications of diabetes, antidiabetic medications, and duration of diabetes | Low | 8 |
| Lee 2011 [62] | Taiwan; population-based | 2000 to 2007; mean 3.9 in metformin users | 20+; 41.9% of metformin user and 48.6% non-users were >60 | 54.9 in metformin users; 54.6 in metformin non-users | Class 1, metformin vs no use of metformin | Colorectal, Liver, and pancreatic cancers | Adjusted for age group, gender, other oral anti-hyperglycemic medication, Charlson comorbidity index score, and duration of metformin exposure which was treated as a time dependent variable | Moderate | 6 |
| Carstensen 2012 [63] | Denmark; population-based | 1995 to 2009; NA | NA | NA | Class 6, insulin vs no use of insulin | Breast, lung, colorectal, prostate, liver, and pancreatic cancer | Adjusted for age, calendar time, and date of birth | Low | 7 |
| Huang 2017 [64] | Taiwan; population-based | 2000 to 2010; mean 6.6 in thiazolidinediones user; 4.7 in nonusers | 20+; 62.1 (14.3) | 52.2 | Class 5, thiazolidinediones vs no use of thiazolidinediones | Liver cancer | Matched on age, sex, and index date;  Adjusted for comorbidities of diabetes, hepatitis B and C, cirrhosis, alcoholic liver disease, NAFLD, end-stage renal disease, hypertension, and hyperlipidemia | Low | 8 |
| Lai 2012b [65] | Taiwan; population-based | 2000 to 2005; NA | 20+; 55.5 (13.4) | 55.8 | Class 1, metformin vs no use of metformin;  Class 3,  alpha-glucosidase inhibitors vs no use of alpha-glucosidase inhibitors;  Class 4, sulfonylureas vs no use of  sulfonylureas  Class 5, thiazolidinediones vs no use of thiazolidinediones;  Class 6, insulins of no use of insulins | Liver cancer | Matched on age and sex;  Adjusted for cirrhosis, alcoholic liver damage, hepatitis B, and hepatitis C | Low | 7 |
| Tseng 2012 [66] | Taiwan; population-based | 2003 to 2005; NA | NA; 77.9% were >40 | 45.3 | Class 1, metformin vs no use of metformin | Colon cancer | Adjusted for age, sex, region of residence, occupation, major comorbidities, diabetes-related complications, other cancer before baseline, commonly used medications, aspirin, nonsteroidal anti-inflammatory drug, anticoagulants, other diabetes medications, and potential colon cancer detection examinations | Low | 7 |
| Tangjarusritaratorn 2021 [67] | Thailand; hospital-based | 2006 to 2019; NA | 20 to 80; 59.0 (9.9) and 60.6 (9.9) in metformin users and nonusers, respectively | 66.6 and 64.3 in metformin users and nonusers, respectively | Class 1, metformin vs no use of metformin | Liver cancer | Adjusted for sex, HbA1c, etiology of cirrhosis, smoke, and alcohol | Low | 7 |
| 25 Case-control studies |  |  |  |  |  |  |  |  |  |
| Bodmer 2012a [68] | United Kingdom; population-based | 1995 to 2009; NA | <90; 69.5 (11.0) | 46.2 | Class 1, metformin vs no prior use of metformin;  Class 4,  sulfonylureas vs no prior use of sulfonylureas;  Class 6, insulin vs no prior use of insulin | Pancreatic cancer | Matched on age, sex, calendar time, general practice, and number of years of active history in the GPRD before the index date;  Adjusted for sulfonylureas, insulin, body mass index, smoking, alcohol consumption, and diabetes duration | Low | 8 |
| Bodmer 2011 [69] | United Kingdom; population-based | 1995 to 2009; NA | NA; 70.2 (8.6) | 63.3 | Class 1, metformin vs no prior use of metformin;  Class 4,  sulfonylureas vs no prior use of sulfonylureas;  Class 6, insulin vs no prior use of insulin | Colorectal cancer | Matched on calendar time (same index date), age (same year of birth), sex, general practice, and number of years of active history in the General Practice Research Database (GPRD) prior to the index date;  Adjusted for each other, body mass index, smoking, diabetes duration, prior use of aspirin, NSAIDs, and statins. | Low | 7 |
| Chang 2012 [70] | Taiwan; population-based | 2000 to 2007; median 7.9 | 30 to 100; 67.0 (10.0) | 64.0 | Class 5,  pioglitazone and rosiglitazone vs no use of thiazolidinediones | Lung, colorectal, and liver cancers | Matched on age (within 5 years), sex, and the number of days of follow-up;  Adjusted for pioglitazone, rosiglitazone, short-acting human insulin, metformin (mean daily dosage in quartiles), sulfonylurea (mean daily dosage in quartiles), number of oral anti-diabetic agents, chronic lung disease, glinides, retinopathy, calcium channel blockers, chronic kidney disease, statins, angiotensin receptor blockers, chronic liver disease, alpha-glucosidase inhibitors | Low | 7 |
| Azoulay 2011 [71] | United Kingdom; population-based | 1988 to 2009; median 3.7 | 40+; 62.7 (11.4) | 100.0 | Class 1, metformin vs never use of metformin | Prostate cancer | Matched on year of birth (+/-1 year), date of cohort entry (+/-1 year);  Adjusted for HbA1c, excessive alcohol use, obesity, smoking, lower urinary tract symptoms, previous cancer, and use of NSAIDs, antihypertensive drugs, statins, and other antidiabetic agents | Low | 8 |
| Bosco 2011 [72] | Denmark; population-based | 1989 to 2008; NA | 50+; 58% of breast cancer cases and 52% of controls were >70 | NA | Class 1, metformin vs no use of metformin | Breast cancer | Conditional logistic regression used to adjust for confounding and selection bias introduced by matching on county and adjusting for complications due to diabetes, clinical obesity, age at index date, postmenopausal hormone use, and multiple imputations to impute missing parity | Moderate | 6 |
| Smiechowski 2013a [73] | United Kingdom; population-based | 1998 to 2009; mean 4.5 | 40+; 72.8 (8.7) in colorectal cancer cases and 72.5 (8.5) in controls | 63.3 in colorectal cancer cases; 63.6 in controls | Class 1, metformin vs no use of metformin | Colorectal cancer | Matched on age (year of birth), sex, calendar year of cohort entry, and duration of follow-up;  Adjusted for obesity, smoking, statins, nonsteroidal anti-inflammatory drugs, aspirin, excessive alcohol use, HbA1c, diabetes duration, cholecystectomy, inflammatory bowel diseases, referrals to colonoscopy, referrals to sigmoidoscopy, history of polyps, previous cancer (other than nonmelanoma skin cancer), and use of sulfonylureas, thiazolidinediones, insulins, and other antidiabetic agents | Low | 7 |
| Margel 2013 [74] | Canada; population-based | 1994 to 2008; NA | 65+; median 76.0 (IQR: 72.0, 80.0) | 100.0 | Class 1, metformin vs no use of metformin;  Class 4,  sulfonylurea no use of sulfonylurea;  Class 5, thiazolidinediones vs no use of thiazolidinediones;  Class 6, insulin vs no use of insulin | Prostate cancer | Matched on age plus year of cohort entry and selected to have the same diabetes duration;  Adjusted for use of other antidiabetic drugs, weighted adjusted clinical groups comorbidity index, socioeconomic status, rural/urban, and use of cyclooxygenase 2, statins and 5-alpha reductase inhibitors (ever vs never) | Low | 7 |
| Chen 2013 [75] | Taiwan; population-based | 1997 to 2008; NA | NA; median 71.2 | 54.6 | Class 5, rosiglitazone and pioglitazone vs no use of thiazolidinediones | Colorectal cancer | Matched on sex, age (i.e., with the same birth calendar year), and the age at the time of diabetes diagnosis (the same calendar year of the initial diabetes diagnosis date);  Adjusted for urbanization, income, colorectal polyp disorder, inflammatory bowel disease, hypertension, hyperlipidemia, biliary stone disease, alcohol-related disease, chronic kidney disease, diabetes-related complications, ischemic heart disease, cerebrovascular disease, heart failure, chronic obstructive pulmonary disease, and hormone therapy | Low | 8 |
| Smiechowski 2013b [76] | United Kingdom; population-based | 1988 to 2009; mean 5.6 | NA; 73.1 (8.5) in lung cancer cases and 73.1 (8.3) in controls | 65.1 | Class 1, metformin vs no use of metformin | Lung cancer | Matched on year of birth (age), sex, calendar year of cohort entry, and duration of follow-up;  Adjusted for diabetes duration, HbA1c, obesity, smoking, excessive alcohol use, previous cancer, chronic obstructive pulmonary disease, asthma, nonsteroidal anti-inflammatory drugs, aspirin, statins, and other antidiabetic drugs | Low | 7 |
| Boxall 2016 [77] | United Kingdom; population-based | 2001 to 2014; mean 5.2 | 40+; 70.3 in prostate cancer cases and 69.5 in controls | 100.0 | Class 1, metformin vs no use of metformin;  Class 4, sulfonylureas vs no use of sulfonylureas;  Class 5, pioglitazone or thiazolidinediones vs no use of pioglitazone or thiazolidinediones;  Class 6, insulins vs no use of insulins | Prostate cancer | Matched on age at cohort entry date ±3 years, cohort entry date ±6 months and region | Low | 7 |
| Rosato 2016 [78] | Italy and Spain; hospital-based and population-based, respectively | 2007 to 2013; NA | Study in Italy:  range 35 to 80, median 67.0 in colorectal cancer cases; range 31 to 80, median 66.0 in controls.  Study in Spain:  range 22 to 85, median 68.0 in colorectal cancer cases; range 28 to 85; median 66.0 in controls. | 65.4 in colorectal cancer cases; 62.6 in controls | Class 1, metformin vs no use of metformin;  Class 4, sulfonylurea derivatives vs no use of sulfonylurea derivatives;  Class 6, insulin vs no use of insulin | Colorectal cancer | Adjusted for study center, sex, age, education, tobacco smoking, alcohol drinking, body mass index, physical activity, statin use, and aspirin use | Moderate | 5 |
| Miele 2015 [79] | Italy; hospital-based | 2005 to 2012; NA | 44.3% of hepatocellular cancer cases and 33.7% of controls were at least 70 | 72.3 in liver cancer cases; 61.7 in controls | Class 1, metformin vs no use of metformin;  Class 4, sulfonylureas vs no use of sulfonylureas;  Class 6, insulin vs no use of insulin | Liver cancer | Age, sex, tobacco smoking, and alcohol drinking | Moderate | 4 |
| Bodmer 2012b [80] | United Kingdom; population-based | 1995 to 2009; NA | <90; 69.0 (10.3) | 58.4 in lung cancer case; 58.5 in controls | Class 1, metformin vs no prior use of metformin;  Class 4, sulfonylureas vs no prior use of Sulfonylureas;  Class 6, insulin vs no prior use of insulin | Lung cancer | Matched on age, sex, calendar time, general practice, and number of years of active history in the General Practice Research Database (GPRD);  Adjusted for Sulfonylureas, insulin, body mass index, and smoking (non-diabetic and diabetic people) | Low | 7 |
| Bosetti 2015 [81] | Italy; population-based | 2000 to 2012; mean 6.0 | NA; 65.2 (9.1) in hepatocellular cancer cases and 65.3 (9.0) in controls | 75.2 | Class 1, metformin vs no use of metformin;  Class 2, incretins vs no use of incretins;  Class 3, alpha-glucosidase inhibitors vs no use of alpha-glucosidase inhibitors;  Class 4, sulfonylureas or repaglinide vs no prior use of Sulfonylureas or repaglinide;  Class 5, thiazolidinediones vs no use of thiazolidinediones;  Class 6, Insulin or insulin analogs vs no use of insulin or insulin analogs | Liver cancer | Matched on sex, age, date at cohort entry, and duration of follow-up;  Adjusted for Charlson comorbidity index, cardio/cerebrovascular diseases, use of antihypertensive drugs, use of antiplatelet drugs, use of statins (in the 5 years before cohort entry), and for use of other antidiabetics (during follow-up) | Low | 7 |
| Sehdev 2015 [82] | United States; hospital-based | 2005 to 2010; NA | Range 26 to 64; mean 57.4 (5.5) in colorectal cancer cases; range 23 to 64; mean 55.23 (5.69) in controls | 59.8 | Class 1, metformin vs no use of metformin;  Class 4, sulfonylurea vs no use of sulfonylurea;  Class 5, thiazolidinediones cs no use of thiazolidinediones;  Class 6, insulin vs no use of insulin | Colorectal cancer | Matched on age, sex, and geographical region;  Adjusted for obesity, polycystic ovary disease, inflammatory bowel disease, sulfonylurea use, coronary artery disease, prescribed nonsteroidal antiinflammatory drugs (NSAIDs), insulin use, thiazolidinedione use, Charlson comorbidity index, number of hospital admissions, and number of outpatient visits | Moderate | 6 |
| Walker 2015 [83] | United States; hospital-based | 2006 to 2011; NA | 21 to 85; 63.0 in pancreatic cancer cases and 60.0 in controls | 53.0 in pancreatic cancer cases; 48.30 in controls | Class 1, metformin to no use of metformin;  Class 4, insulin secretagogues vs no use of insulin secretagogues;  Class 5, thiazolidinediones cs no use of thiazolidinediones;  Class 6, insulin vs no use of insulin | Pancreatic cancer | Adjusted for age, sex and other classes of diabetes drugs (insulin secretagogues, thiazolidinediones, and insulin) | Moderate | 4 |
| Azoulay 2016 [84] | Canada, United States, United Kingdom; population-based | 2007 to 2013; NA | 19+; 68.20 in pancreatic cancer cases and 68.10 in controls | 50.9 | Class 2, dipeptidyl peptidase-4 inhibitors (linagliptin, sitagliptin, vildagliptin, saxagliptin) or glucagon-like peptide-1 receptor agonists (exenatide, liraglutide) vs use of other diabetes medication(s) | Pancreatic cancer | Matched on age (365 days either way), sex, date of entry to the study cohort (180 days either way), duration of treated diabetes before entry to the study cohort (defined as time between entry to the base cohort and entry to the study cohort; 90 days either way), and duration of follow-up;  Adjusted for alcohol related disorders, history of acute or chronic pancreatitis, microvascular complications of diabetes (neuropathy, renal disease, retinopathy, and peripheral arteriopathy), number of hospital admissions, number of unique non-diabetic drugs in previous year, number of antidiabetic drugs received before entry to study cohort, ever use of statins.  In the clinical practice research datalink, models were further adjusted for body mass index, smoking status, and glycated hemoglobin a1c level (<7. 0 % (53 mmol/mol), 7.1-8.0% (54-64 mmol/mol), >8.0% (64 mmol/mol)) | Low | 7 |
| Lu 2015 [85] | United Kingdom; population-based | 1996 to 2010; NA | 20 to 79; ~75% were > 65 | 58.0 in pancreatic cancer cases; 57.1 in controls | Class 1, metformin vs no use of metformin;  Class 4, sulfonylureas vs no use of sulfonylureas;  Class 5, glitazones vs no use of glitazones;  Class 6, insulin vs no use of insulin | Pancreatic cancer | Adjusted for age, sex, body mass index, smoking, alcohol drinking, Townsend deprivation index, calendar year, GP visit 1 year before index date, and diabetes | Low | 8 |
| Feng 2015 [86] | Multicenter; hospital-based | NA (4 years); NA | 58 to 70; median 64.0 (IQR: 59.0, 68.0) in people taking metformin | 100.0 | Class 1, metformin vs no use of metformin | Prostate cancer | Adjusted for age, race, geographic region, prostate specific antigen levels, digital rectal examination findings, body mass index, prostate volume, family history of prostate cancer, coronary artery disease, smoking status, NSAIDs, statins, aspirin, and treatment group | Low | 7 |
| Cardel 2014 [87] | Denmark; population-based | 2000 to 2004 and 2005 to 2009; NA | NA; median 73.0 (IQR: 66.0, 80.0) in colorectal cancer cases and 69.0 (IQR: 61.0, 77.0) in controls | 55.0 | Class 1, metformin vs no use of metformin | Colorectal cancer | Adjusted for age, gender, calendar year, tobacco, obesity, alcohol use, aspirin, NSAIDs, statins, glibenclamide, and gliclazide | Low | 7 |
| Liao 2019 [88] | Taiwan; population-based | 2000 to 2011; NA | 20 to 84; 68.2 (9.7) in colorectal cancer cases and 68.1 (9.6) in controls | 56.0 in colorectal cancer cases; 55.2 in controls | Class 5, thiazolidinediones (pioglitazone and rosiglitazone) vs no use of thiazolidinediones | Colorectal cancer | Adjusted for other anti-diabetic drugs and alcohol-related disease | Moderate | 6 |
| Shin 2020 [89] | South Korea; population-based | 2007 to 2014; NA | 30+; 61.3 (9.5) in colorectal cancer cases and 61.4 (9.1) in controls | 65.3 in colorectal cancer cases; 23.5 in controls | Class 1, metformin vs no use of metformin;  Class 2, dipeptidyl peptidase-4 inhibitor vs no use of dipeptidyl peptidase-4 inhibitor;  Class 3, alpha-glucosidase inhibitor vs no use of alpha-glucosidase inhibitor;  Class 4, sulfonylurea or meglitinide vs no use of sulfonylurea or meglitinide;  Class 5, thiazolidinedione vs no use of thiazolidinedione;  Class 6, insulin vs no use of insulin | Colorectal cancer | Matched on age, sex, and the year of diagnosis of type 2 diabetes;  Adjusted for smoking, drinking, waist circumference, exercise, insulin use, and the time from diabetes mellitus diagnosis to index date | Low | 7 |
| Lee 2019b [90] | South Korea; population-based | 2002 to 2013; NA | 40+; >50% were 60+ | 80.5 | Class 4, sulfonylurea vs never use of sulfonylurea | Liver cancer | Matched on the age at the index date (defined as the date 1 year before the Hepatocellular Carcinoma diagnosis), sex, and the year of the diabetes diagnosis;  Adjusted for alcoholic liver disease, chronic viral hepatitis, liver cirrhosis, chronic lower respiratory disease, history of previous cancer, Charlson comorbidity index, household income level, residential area, aspirin use, statin use, and other anti-diabetic agent (insulin, glinide, metformin, thiazolidinedione, dipeptidyl depeptidase-4 inhibitor) use | Moderate | 5 |
| Demb 2019 [91] | United States; US Veterans | NA; NA | NA; median 69.0 (IQR: 62.0, 76.0) in colorectal cancer cases and 63.0 (IQR: 58.0, 70.0) in controls; | 99 in colorectal cancer cases; 97 in controls | Class 1, metformin vs no use of metformin | Colorectal cancer | Adjusted for Age, sex, race/ethnicity, body mass index, smoking status, and aspirin exposure | Moderate | 6 |
| Hosio 2019 [92] | Finland; population-based | 1996 to 2011; mean 5.4 | 40+; ~50% aged > 70 | NA | Class 1, metformin vs no use of metformin;  Class 6, insulin vs no use of insulin | Breast cancer | Matched on age and duration of diabetes | Moderate | 6 |

**Supplementary references**

1. Hernandez A.F. *et al.* Albiglutide and cardiovascular outcomes in patients with type 2 diabetes and cardiovascular disease (Harmony Outcomes): a double-blind, randomised placebo-controlled trial. *The Lancet* **392,** 1519–1529 (2018).

2. Jones, N. P., Curtis, P. S. & Home, P. D. Cancer and bone fractures in observational follow-up of the RECORD study. *Acta diabetologica* **52,** 539–46 (2015).

3. Bethel, M. A. *et al.* Assessing the Safety of Sitagliptin in Older Participants in the Trial Evaluating Cardiovascular Outcomes with Sitagliptin (TECOS). *Diabetes care* **40,** 494–501 (2017).

4. Soffer, D. *et al.* Metformin and breast and gynecological cancer risk among women with diabetes. *BMJ Open Diabetes Research & Care* **3,** 9 (2015).

5. Tsai, M. J. *et al.* Metformin decreases lung cancer risk in diabetic patients in a dose-dependent manner. *Lung Cancer* **86,** 137–143 (2014).

6. Habel L.A. *et al.* Cohort study of insulin glargine and risk of breast, prostate, and colorectal cancer among patientswith diabetes. *Diabetes Care* **36,** 3953–3960 (2013).

7. Knapen L.M. *et al.* Use of incretin agents and risk of pancreatic cancer: A population-based cohort study. *Diabetes, Obesity and Metabolism* **18,** 258–265 (2016).

8. Funch D. *et al.* Is there an association between liraglutide use and female breast cancer in a real-world setting? *Diabetes, Metabolic Syndrome and Obesity: Targets and Therapy* **11,** 791–806 (2018).

9. Chang, C.-H. *et al.* Cancer risk associated with insulin glargine among adult type 2 diabetes patients--a nationwide cohort study. *PloS one* **6,** e21368 (2011).

10. Suissa, S. *et al.* Long-term effects of insulin glargine on the risk of breast cancer. *Diabetologia* **54,** 2254 (2011).

11. Lai, S.-W. *et al.* Antidiabetes drugs correlate with decreased risk of lung cancer: a population-based observation in Taiwan. *Clinical lung cancer* **13,** 143–8 (2012).

12. Funch, D., Gydesen, H., Tornoe, K., Major-Pedersen, A. & Chan, K. A. A prospective, claims-based assessment of the risk of pancreatitis and pancreatic cancer with liraglutide compared to other antidiabetic drugs. *Diabetes, obesity & metabolism* **16,** 273–5 (2014).

13. Tseng, C.-H. Human insulin does not increase prostate cancer risk in Taiwanese. *Clinical genitourinary cancer* **12,** e7-12 (2014).

14. Vallarino, C. *et al.* Comparing pioglitazone to insulin with respect to cancer, cardiovascular and bone fracture endpoints, using propensity score weights. *Clinical drug investigation* **33,** 621–31 (2013).

15. Schlesinger, S. *et al.* Diabetes mellitus, insulin treatment, diabetes duration, and risk of biliary tract cancer and hepatocellular carcinoma in a European cohort. *Annals of oncology : official journal of the European Society for Medical Oncology* **24,** 2449–55 (2013).

16. Gu, Y. *et al.* Cancer incidence and mortality in patients with type 2 diabetes treated with human insulin: a cohort study in Shanghai. *PloS one* **8,** e53411 (2013).

17. Kao, C.-H. *et al.* A population-based cohort study in Taiwan--use of insulin sensitizers can decrease cancer risk in diabetic patients? *Annals of oncology : official journal of the European Society for Medical Oncology* **24,** 523–530 (2013).

18. Fagot, J.-P. *et al.* Does insulin glargine increase the risk of cancer compared with other basal insulins?: A French nationwide cohort study based on national administrative databases. *Diabetes care* **36,** 294–301 (2013).

19. Redaniel, M. T. M., Jeffreys, M., May, M. T., Ben-Shlomo, Y. & Martin, R. M. Associations of type 2 diabetes and diabetes treatment with breast cancer risk and mortality: a population-based cohort study among British women. *Cancer causes & control : CCC* **23,** 1785–95 (2012).

20. Sakoda, L. C. *et al.* Metformin use and lung cancer risk in patients with diabetes. *Cancer prevention research (Philadelphia, Pa.)* **8,** 174–9 (2015).

21. Calip, G. S., Yu, O., Elmore, J. G. & Boudreau, D. M. Comparative safety of diabetes medications and risk of incident invasive breast cancer: a population-based cohort study. *Cancer causes & control: CCC* **27,** 709–20 (2016).

22. Wang, C.-P. *et al.* Metformin for Reducing Racial/Ethnic Difference in Prostate Cancer Incidence for Men with Type II Diabetes. *Cancer prevention research (Philadelphia, Pa.)* **9,** 779–787 (2016).

23. Erdmann, E., Harding, S., Lam, H. & Perez, A. Ten-year observational follow-up of PROactive: a randomized cardiovascular outcomes trial evaluating pioglitazone in type 2 diabetes. *Diabetes, obesity & metabolism* **18,** 266–73 (2016).

24. Tseng, C.-H. Sitagliptin and pancreatic cancer risk in patients with type 2 diabetes. *European journal of clinical investigation* **46,** 70–9 (2016).

25. Tseng, C.-H. Prolonged use of human insulin increases breast cancer risk in Taiwanese women with type 2 diabetes. *BMC cancer* **15,** 846 (2015).

26. Tuccori, M., Wu, J. W., Yin, H., Majdan, A. & Azoulay, L. The Use of Glyburide Compared With Other Sulfonylureas and the Risk of Cancer in Patients With Type 2 Diabetes. *Diabetes care* **38,** 2083–9 (2015).

27. Tseng, C.-H. Metformin significantly reduces incident prostate cancer risk in Taiwanese men with type 2 diabetes mellitus. *European journal of cancer (Oxford, England : 1990)* **50,** 2831–7 (2014).

28. Gokhale, M. *et al.* Dipeptidyl-peptidase-4 inhibitors and pancreatic cancer: a cohort study. *Diabetes, obesity & metabolism* **16,** 1247–56 (2014).

29. Tsilidis, K. K. *et al.* Metformin does not affect cancer risk: a cohort study in the U.K. Clinical Practice Research Datalink analyzed like an intention-to-treat trial. *Diabetes care* **37,** 2522–32 (2014).

30. Tseng, C.-H. Metformin may reduce breast cancer risk in Taiwanese women with type 2 diabetes. *Breast cancer research and treatment* **145,** 785–90 (2014).

31. Vicentini, M. *et al.* Effect of different glucose-lowering therapies on cancer incidence in type 2 diabetes: An observational population-based study. *Diabetes research and clinical practice* **143,** 398–408 (2018).

32. Bradley, M. C. *et al.* A Cohort Study of Metformin and Colorectal Cancer Risk among Patients with Diabetes Mellitus. *Cancer epidemiology, biomarkers & prevention : a publication of the American Association for Cancer Research, cosponsored by the American Society of Preventive Oncology* **27,** 525–530 (2018).

33. Tseng, C.-H. Metformin and lung cancer risk in patients with type 2 diabetes mellitus. *Oncotarget* **8,** 41132–41142 (2017).

34. Tseng, C.-H. Metformin is associated with a lower risk of colorectal cancer in Taiwanese patients with type 2 diabetes: A retrospective cohort analysis. *Diabetes & metabolism* **43,** 438–445 (2017).

35. Abrahami, D., Yin, H., Yu, O. H. Y., Pollak, M. N. & Azoulay, L. Incretin-based Drugs and the Incidence of Colorectal Cancer in Patients with Type 2 Diabetes. *Epidemiology (Cambridge, Mass.)* **29,** 246–253 (2018).

36. Boniol, M. *et al.* Incretin-Based Therapies and the Short-term Risk of Pancreatic Cancer: Results From Two Retrospective Cohort Studies. *Diabetes care* **41,** 286–292 (2018).

37. Tseng, C.-H. Pioglitazone and lung cancer risk in Taiwanese patients with type 2 diabetes. *Diabetes & metabolism* **44,** 77–79 (2018).

38. You, J. H. *et al.* Metformin and Gastrointestinal Cancer Development in Newly Diagnosed Type 2 Diabetes: A Population-Based Study in Korea. *Clinical and translational gastroenterology* **11,** e00254 (2020).

39. Rouette, J. *et al.* Incretin-based drugs and risk of lung cancer among individuals with type 2 diabetes. *Diabetic medicine : a journal of the British Diabetic Association* **37,** 868–875 (2020).

40. Haring, A. *et al.* Antidiabetic drug use and prostate cancer risk in the Finnish Randomized Study of Screening for Prostate Cancer. *Scandinavian journal of urology* **51,** 5–12 (2017).

41. Chen, C. B., Eurich, D. T., Majumdar, S. R. & Johnson, J. A. Metformin and the risk of prostate cancer across racial/ethnic groups: a population-based cohort study. *Prostate cancer and prostatic diseases* **20,** 122–126 (2017).

42. Tseng, C.-H. Sitagliptin May Reduce Breast Cancer Risk in Women With Type 2 Diabetes. *Clinical breast cancer* **17,** 211–218 (2017).

43. Tseng, C.-H. Rosiglitazone reduces breast cancer risk in Taiwanese female patients with type 2 diabetes mellitus. *Oncotarget* **8,** 3042–3048 (2017).

44. Bowker, S. L., Lin, M., Eurich, D. T. & Johnson, J. A. Time-Varying Risk for Breast Cancer Following Initiation of Glucose-Lowering Therapy in Women with Type 2 Diabetes: Exploring Detection Bias. *Canadian journal of diabetes* **41,** 204–210 (2017).

45. Tseng, C.-M. *et al.* Incretin-based pharmacotherapy and risk of adverse pancreatic events in the ethnic Chinese with diabetes mellitus: A population-based study in Taiwan. *Pancreatology : official journal of the International Association of Pancreatology (IAP) ... [et al.]* **17,** 76–82 (2017).

46. Tseng, C.-H. Sitagliptin may reduce prostate cancer risk in male patients with type 2 diabetes. *Oncotarget* **8,** 19057–19064 (2017).

47. Tseng, C.-H. Human Insulin Therapy Is Associated With an Increased Risk of Lung Cancer: A Population-Based Retrospective Cohort Study. *Frontiers in endocrinology* **10,** 443 (2019).

48. Erkinantti, S. *et al.* Association of Metformin, Other Antidiabetic Medications, and Statins With Incidence of Colon Cancer in Patients With Type 2 Diabetes. *Clinical colorectal cancer* (2020). doi:10.1016/j.clcc.2020.11.003

49. Lee, M. *et al.* Nationwide Trends in Pancreatitis and Pancreatic Cancer Risk Among Patients With Newly Diagnosed Type 2 Diabetes Receiving Dipeptidyl Peptidase 4 Inhibitors. *Diabetes care* **42,** 2057–2064 (2019).

50. Liang, C. *et al.* Exenatide use and incidence of pancreatic and thyroid cancer: A retrospective cohort study. *Diabetes, obesity & metabolism* **21,** 1037–1042 (2019).

51. Chang, Y.-T. *et al.* Dose-Dependent Relationship Between Metformin and Colorectal Cancer Occurrence Among Patients with Type 2 Diabetes-A Nationwide Cohort Study. *Translational oncology* **11,** 535–541 (2018).

52. Albai, O. *et al.* Long-term Risk of Malignant Neoplastic Disorders in Type 2 Diabetes Mellitus Patients with Metabolic Syndrome. *Diabetes, metabolic syndrome and obesity : targets and therapy* **13,** 1317–1326 (2020).

53. Yen, F.-S., Wei, J. C.-C., Yang, Y.-C., Hsu, C.-C. & Hwu, C.-M. Respiratory outcomes of metformin use in patients with type 2 diabetes and chronic obstructive pulmonary disease. *Scientific reports* **10,** 10298 (2020).

54. Bradley, M. C. *et al.* Similar Breast Cancer Risk in Women Older Than 65 Years Initiating Glargine, Detemir, and NPH Insulins. *Diabetes care* **43,** 785–792 (2020).

55. Chen, C.-H., Lin, C.-L., Hsu, C.-Y. & Kao, C.-H. Insulin enhances and metformin reduces risk of colorectal carcinoma in type-2 diabetes. *QJM : monthly journal of the Association of Physicians* **113,** 194–200 (2020).

56. Wang, C.-P. *et al.* Metformin modifies disparity in hepatocellular carcinoma incidence in men with type 2 diabetes but without chronic liver diseases. *Cancer medicine* **8,** 3206–3215 (2019).

57. Kincius, M., Patasius, A., Linkeviciute-Ulinskiene, D., Zabuliene, L. & Smailyte, G. Reduced risk of prostate cancer in a cohort of Lithuanian diabetes mellitus patients. *The aging male : the official journal of the International Society for the Study of the Aging Male* 1–6 (2020). doi:10.1080/13685538.2020.1766013

58. Yen, F.-S. *et al.* Dipeptidyl peptidase-4 inhibitors may accelerate cirrhosis decompensation in patients with diabetes and liver cirrhosis: a nationwide population-based cohort study in Taiwan. *Hepatology international* **15,** 179–190 (2021).

59. Tseng, C.-H. Metformin and risk of hepatocellular carcinoma in patients with type 2 diabetes. *Liver international : official journal of the International Association for the Study of the Liver* **38,** 2018–2027 (2018).

60. Lee, D. Y. *et al.* The influence of diabetes and antidiabetic medications on the risk of pancreatic cancer: a nationwide population-based study in Korea. *Scientific reports* **8,** 9719 (2018).

61. Lee, J. M., Lee, K.-M., Kim, D. B., Ko, S.-H. & Park, Y. G. Colorectal Cancer Risks According to Sex Differences in Patients With Type II Diabetes Mellitus: A Korean Nationwide Population-Based Cohort Study. *Clinical and translational gastroenterology* **10,** e00090 (2019).

62. Lee, M.-S. *et al.* Type 2 diabetes increases and metformin reduces total, colorectal, liver and pancreatic cancer incidences in Taiwanese: a representative population prospective cohort study of 800,000 individuals. *BMC cancer* **11,** 20 (2011).

63. Carstensen, B., Witte, D. R. & Friis, S. Cancer occurrence in Danish diabetic patients: duration and insulin effects. *Diabetologia* **55,** 948–58 (2012).

64. Huang, M.-Y. *et al.* The role of thiazolidinediones in hepatocellular carcinoma risk reduction: a population-based cohort study in Taiwan. *American journal of cancer research* **7,** 1606–1616 (2017).

65. Lai, S.-W. *et al.* Risk of Hepatocellular Carcinoma in Diabetic Patients and Risk Reduction Associated With Anti-Diabetic Therapy: A Population-Based Cohort Study. *American Journal of Gastroenterology* **107,** 46–52 (2012).

66. Tseng, C.-H. Diabetes, metformin use, and colon cancer: a population-based cohort study in Taiwan. *European journal of endocrinology* **167,** 409–16 (2012).

67. Tangjarusritaratorn, T., Tangjittipokin, W. & Kunavisarut, T. Incidence and Survival of Hepatocellular Carcinoma in Type 2 Diabetes Patients with Cirrhosis Who Were Treated with and without Metformin. *Diabetes Metab Syndr Obes* **14,** 1563–1574 (2021).

68. Bodmer, M., Becker, C., Meier, C., Jick, S. S. & Meier, C. R. Use of antidiabetic agents and the risk of pancreatic cancer: a case-control analysis. *The American journal of gastroenterology* **107,** 620–6 (2012).

69. Bodmer, M., Becker, C., Meier, C., Jick, S. S. & Meier, C. R. Use of metformin is not associated with a decreased risk of colorectal cancer: a case-control analysis. *Cancer epidemiology, biomarkers & prevention : a publication of the American Association for Cancer Research, cosponsored by the American Society of Preventive Oncology* **21,** 280–6 (2012).

70. Chang, C.-H. *et al.* Association of thiazolidinediones with liver cancer and colorectal cancer in type 2 diabetes mellitus. *Hepatology (Baltimore, Md.)* **55,** 1462–72 (2012).

71. Azoulay, L., Dell’Aniello, S., Gagnon, B., Pollak, M. & Suissa, S. Metformin and the incidence of prostate cancer in patients with type 2 diabetes. *Cancer epidemiology, biomarkers & prevention : a publication of the American Association for Cancer Research, cosponsored by the American Society of Preventive Oncology* **20,** 337–44 (2011).

72. Bosco, J. L. F., Antonsen, S., Sorensen, H. T., Pedersen, L. & Lash, T. L. Metformin and incident breast cancer among diabetic women: a population-based case-control study in Denmark. *Cancer epidemiology, biomarkers & prevention : a publication of the American Association for Cancer Research, cosponsored by the American Society of Preventive Oncology* **20,** 101–11 (2011).

73. Smiechowski, B., Azoulay, L., Yin, H., Pollak, M. N. & Suissa, S. The use of metformin and colorectal cancer incidence in patients with type II diabetes mellitus. *Cancer epidemiology, biomarkers & prevention : a publication of the American Association for Cancer Research, cosponsored by the American Society of Preventive Oncology* **22,** 1877–83 (2013).

74. Margel, D. *et al.* Association between metformin use and risk of prostate cancer and its grade. *Journal of the National Cancer Institute* **105,** 1123–31 (2013).

75. Chen, S.-W. *et al.* Use of thiazolidinediones and the risk of colorectal cancer in patients with diabetes: a nationwide, population-based, case-control study. *Diabetes care* **36,** 369–75 (2013).

76. Smiechowski, B. B., Azoulay, L., Yin, H., Pollak, M. N. & Suissa, S. The use of metformin and the incidence of lung cancer in patients with type 2 diabetes. *Diabetes care* **36,** 124–9 (2013).

77. Boxall, N., Bennett, D., Hunger, M., Dolin, P. & Thompson, P. L. Evaluation of exposure to pioglitazone and risk of prostate cancer: a nested case-control study. *BMJ open diabetes research & care* **4,** e000303 (2016).

78. Rosato, V. *et al.* Type 2 Diabetes, Antidiabetic Medications, and Colorectal Cancer Risk: Two Case-Control Studies from Italy and Spain. *Frontiers in oncology* **6,** 210 (2016).

79. Miele, L. *et al.* Diabetes and Insulin Therapy, but Not Metformin, Are Related to Hepatocellular Cancer Risk. *Gastroenterology research and practice* **2015,** 570356 (2015).

80. Bodmer, M., Becker, C., Jick, S. S. & Meier, C. R. Metformin does not alter the risk of lung cancer: a case-control analysis. *Lung cancer (Amsterdam, Netherlands)* **78,** 133–7 (2012).

81. Bosetti, C. *et al.* Insulin and other antidiabetic drugs and hepatocellular carcinoma risk: a nested case-control study based on Italian healthcare utilization databases. *Pharmacoepidemiology and drug safety* **24,** 771–8 (2015).

82. Sehdev, A. *et al.* Metformin for primary colorectal cancer prevention in patients with diabetes: a case-control study in a US population. *Cancer* **121,** 1071–8 (2015).

83. Walker, E. J., Ko, A. H., Holly, E. A. & Bracci, P. M. Metformin use among type 2 diabetics and risk of pancreatic cancer in a clinic-based case-control study. *International journal of cancer* **136,** E646-53 (2015).

84. Azoulay, L. *et al.* Incretin based drugs and the risk of pancreatic cancer: International multicentre cohort study. *BMJ* **352,** (2016).

85. Lu, Y. *et al.* New-onset type 2 diabetes, elevated HbA1c, anti-diabetic medications, and risk of pancreatic cancer. *British journal of cancer* **113,** 1607–14 (2015).

86. Feng, T. *et al.* Metformin use and risk of prostate cancer: results from the REDUCE study. *Cancer prevention research (Philadelphia, Pa.)* **8,** 1055–60 (2015).

87. Cardel, M., Jensen, S. M., Pottegard, A., Jorgensen, T. L. & Hallas, J. Long-term use of metformin and colorectal cancer risk in type II diabetics: a population-based case-control study. *Cancer medicine* **3,** 1458–66 (2014).

88. Liao, K.-F., Lin, C.-L. & Lai, S.-W. Association between colorectal cancer and thiazolidinediones administration in a case-control study. *BioMedicine* **9,** 4 (2019).

89. Shin, C. M. *et al.* Anti-diabetic medications and the risk for colorectal cancer: A population-based nested case-control study. *Cancer epidemiology* **64,** 101658 (2020).

90. Lee, J.-Y., Jang, S.-Y., Nam, C. M. & Kang, E. S. Incident Hepatocellular Carcinoma Risk in Patients Treated with a Sulfonylurea: A Nationwide, Nested, Case-Control Study. *Scientific reports* **9,** 8532 (2019).

91. Demb, J. *et al.* Metformin Is Associated With Reduced Odds for Colorectal Cancer Among Persons With Diabetes. *Clinical and translational gastroenterology* **10,** e00092 (2019).

92. Hosio, M. *et al.* Association of antidiabetic medication and statins with breast cancer incidence in women with type 2 diabetes. *Breast Cancer Res Treat* **175,** 741–748 (2019).
