# Supplementary material for: Diabetes medications and cancer risk associations: a systematic review and meta-analysis of evidence over the past 10 years
Source: Sci Rep. 2023 Jul 22;13:11844. doi: 10.1038/s41598-023-38431-z (PMC10363143; doi:10.1038/s41598-023-38431-z)
Supplement: Supplementary file 2 — Supplementary Information 2. [file 41598_2023_38431_MOESM2_ESM.docx]

**Diabetes medications and cancer risk associations: a systematic review and meta-analysis of evidence over the past 10 years**

**Author Details**

* Yixian Chen: School of Population and Public Health, University of British Columbia, Vancouver, Canada. ychen153@student.ubc.ca

Fidela Mushashi: BC Cancer, Vancouver, Canada. fidela.mushashi@bccancer.bc.ca

Surim Son: Department of Epidemiology and Biostatistics, Schulich School of Medicine & Dentistry, Western University, London, Ontario, Canada. sson8@uwo.ca

Parveen Bhatti: Cancer Control Research, BC Cancer and School of Population and Public Health, University of British Columbia, Vancouver, Canada. pbhatti@bccrc.ca

Trevor Dummer: School of Population and Public Health, University of British Columbia, Vancouver, Canada. trevor.dummer@ubc.ca

Rachel A. Murphy: Cancer Control Research, BC Cancer and School of Population and Public Health, University of British Columbia, Vancouver, Canada. rachel.murphy@ubc.ca

**Corresponding Author**

Yixian Chen: School of Population and Public Health, University of British Columbia, Vancouver, Canada. ychen153@student.ubc.ca

Mailing address: Rm 167-2206 East Mall, Vancouver, BC V6T 1Z3, Canada

**SUPPLEMENTARY FILE 2**

**Supplementary Figure 1. Pooled analysis of the association between biguanides and breast cancer risk**

**
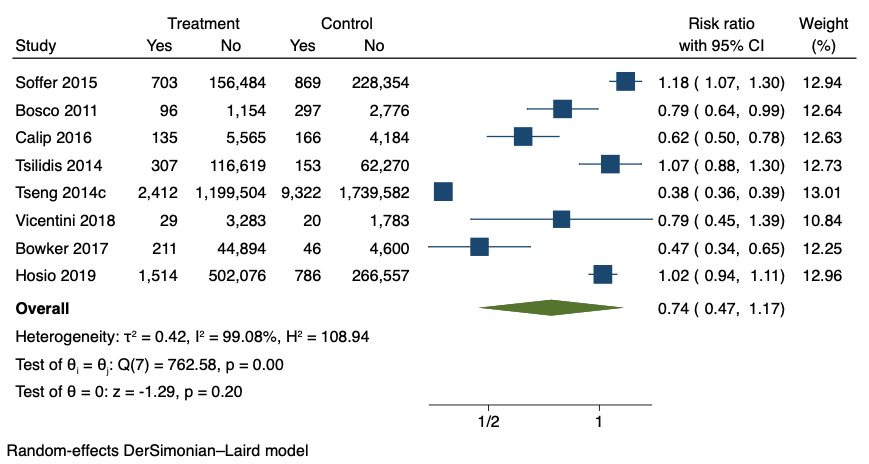
**

**Supplementary Figure 2. Pooled analysis of the association between incretin-based medicines and breast cancer risk**

**
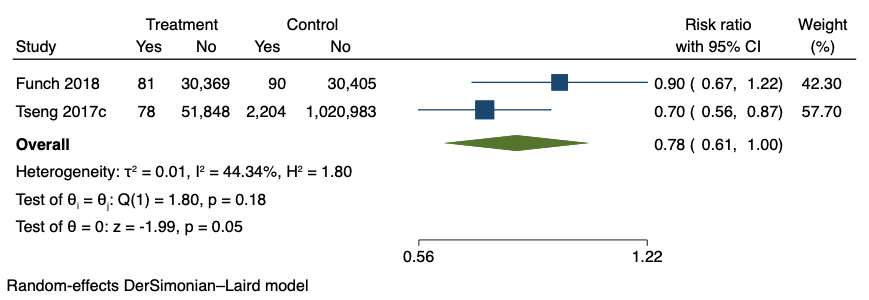
**

**Supplementary Figure 3. Pooled analysis of the association between insulin secretagogues and breast cancer risk**

**
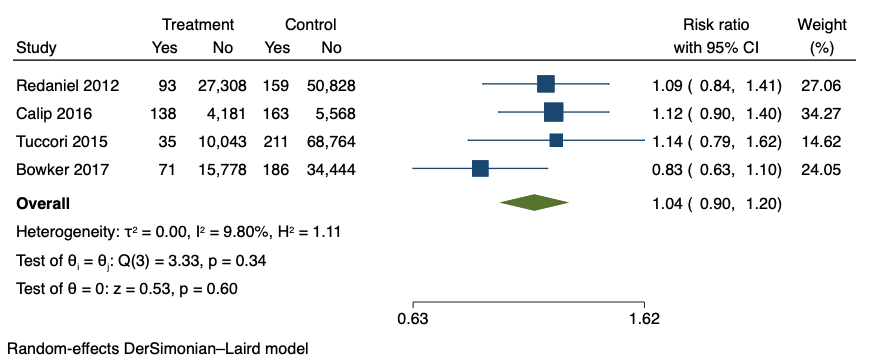
**

**Supplementary Figure 4. Pooled analysis of the association between thiazolidinediones and breast cancer risk**

**
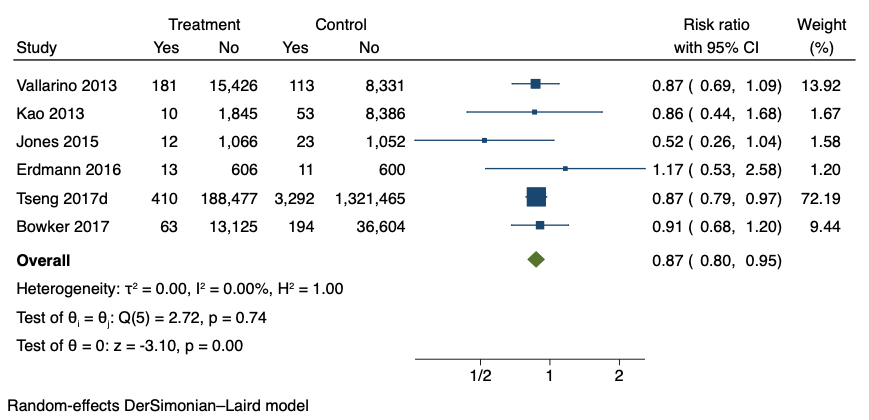
**

**Supplementary Figure 5. Pooled analysis of the association between insulins and breast cancer risk**

**
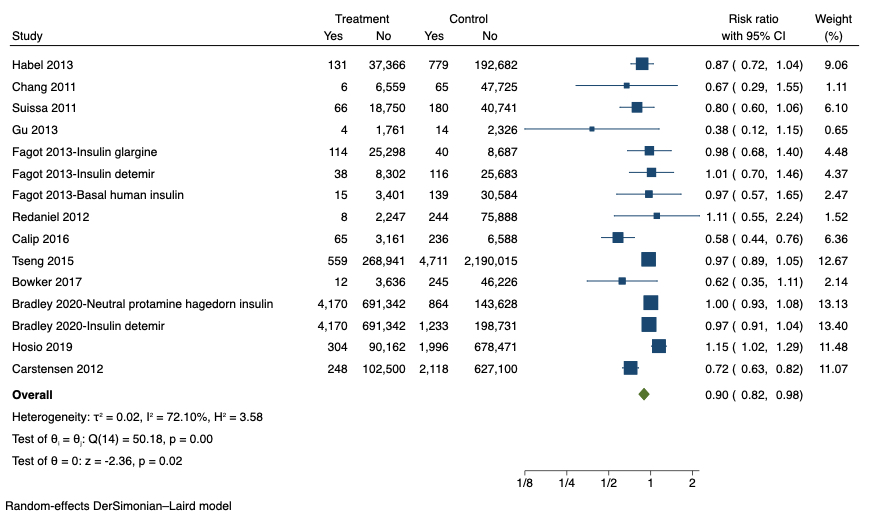
**

**Supplementary Figure 6. Pooled analysis of the association between biguanides and lung cancer risk**

**
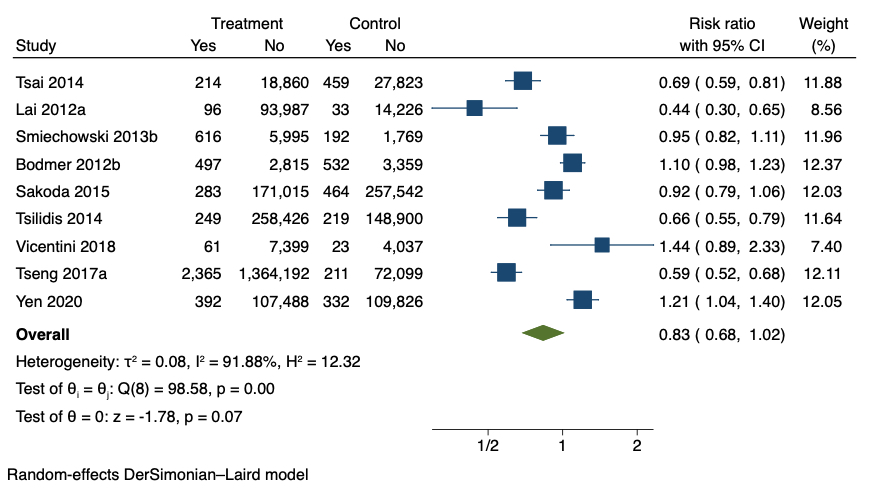
**

**Supplementary Figure 7. Pooled analysis of the association between incretin-based medicines and lung cancer risk**

**
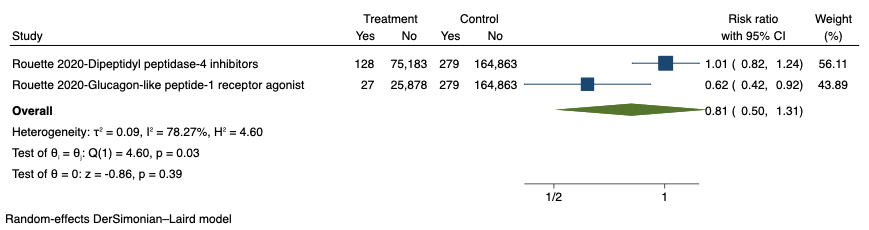
**

**Supplementary Figure 8. Pooled analysis of the association between insulin secretagogues and lung cancer risk**

**
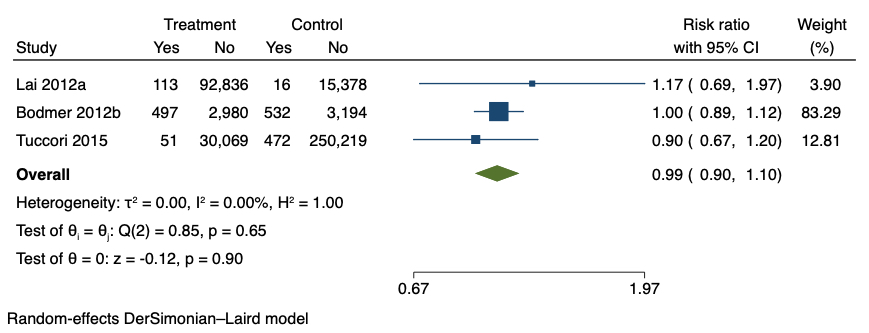
**

**Supplementary Figure 9. Pooled analysis of the association between thiazolidinediones and lung cancer risk**

**
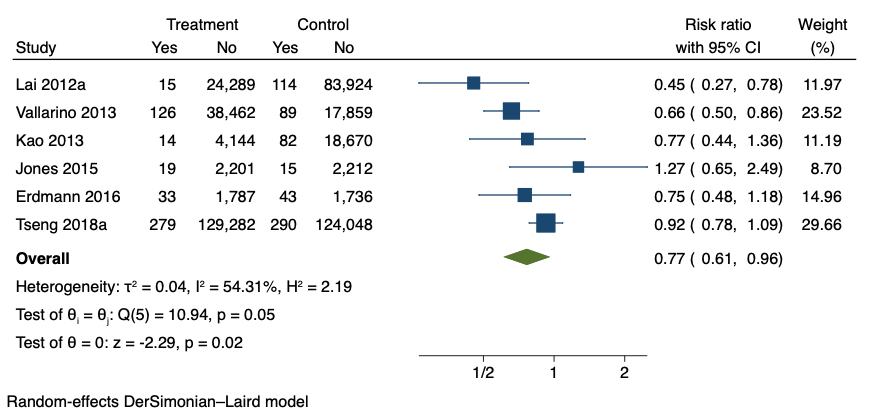
**

**Supplementary Figure 10. Pooled analysis of the association between insulins and lung cancer risk**

**
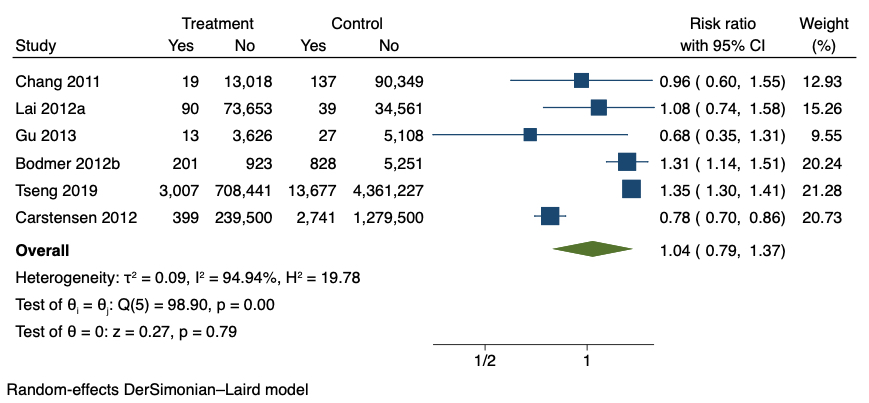
**

**Supplementary Figure 11. Pooled analysis of the association between biguanides and colorectal cancer risk**

**
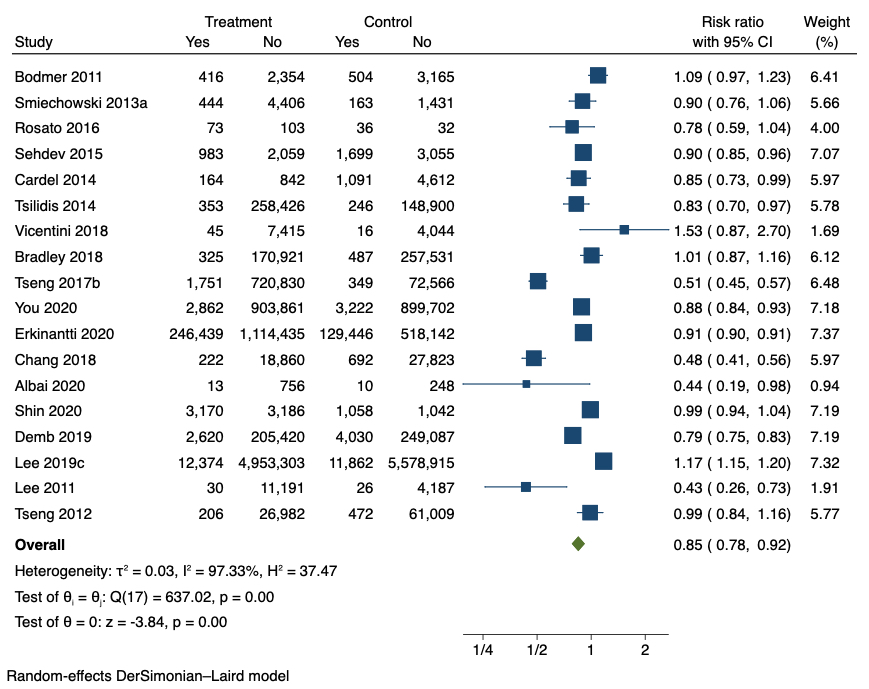
**

**Supplementary Figure 12. Pooled analysis of the association between incretin-based medicines and colorectal cancer risk**

**
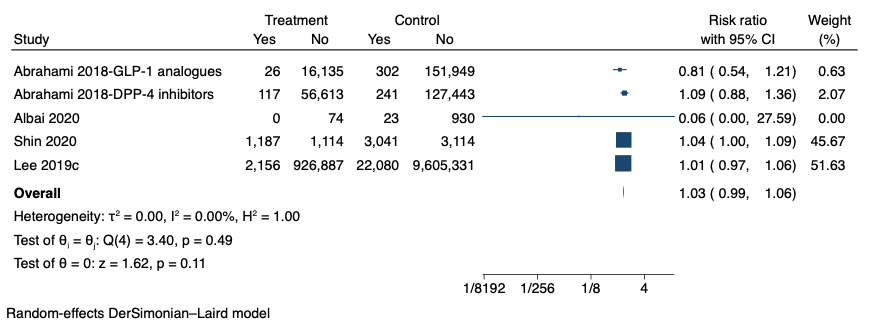
**

**Supplementary Figure 13. Pooled analysis of the association between alpha-glucosidase inhibitors and colorectal cancer risk**

**
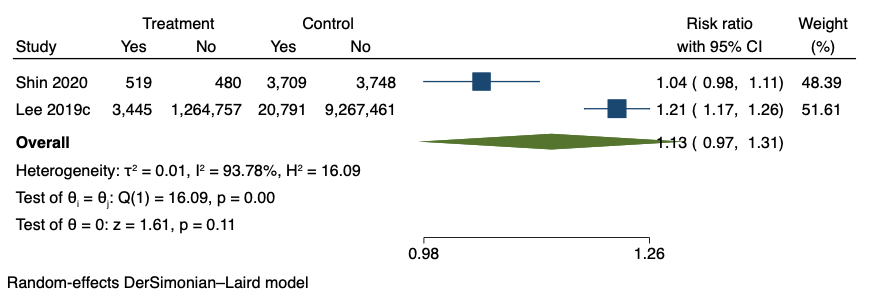
**

**Supplementary Figure 14. Pooled analysis of the association between insulin secretagogues and colorectal cancer risk**

**
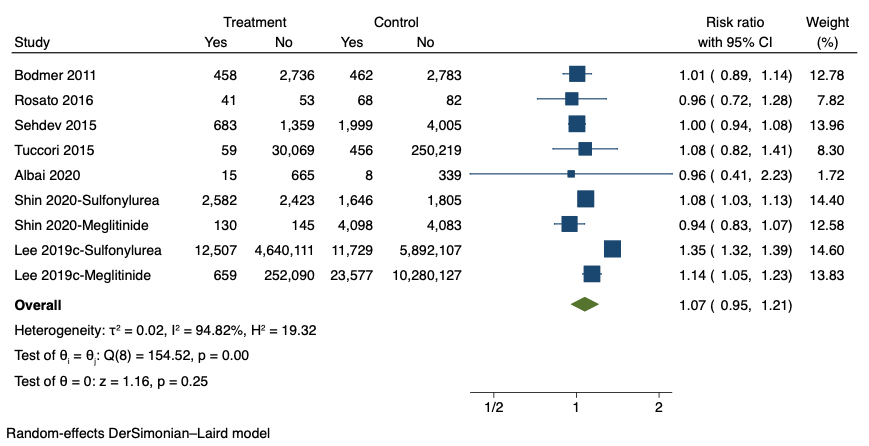
**

**Supplementary Figure 15. Pooled analysis of the association between thiazolidinediones and colorectal cancer risk**

**
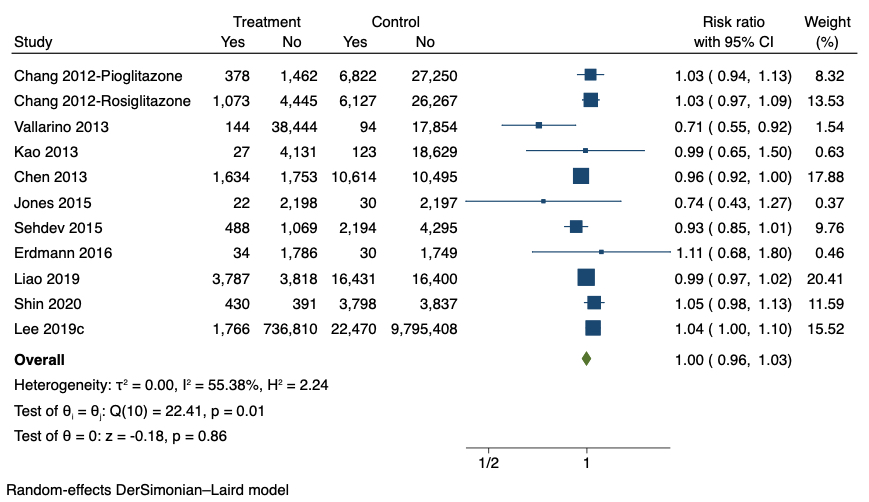
**

**Supplementary Figure 16. Pooled analysis of the association between insulins and colorectal cancer risk**

**
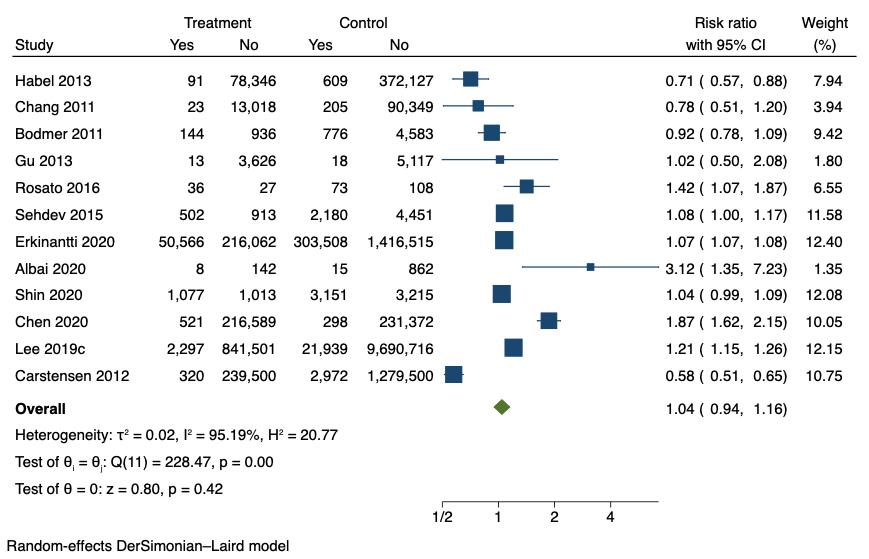
**

**Supplementary Figure 17. Pooled analysis of the association between biguanides and prostate cancer risk**

**
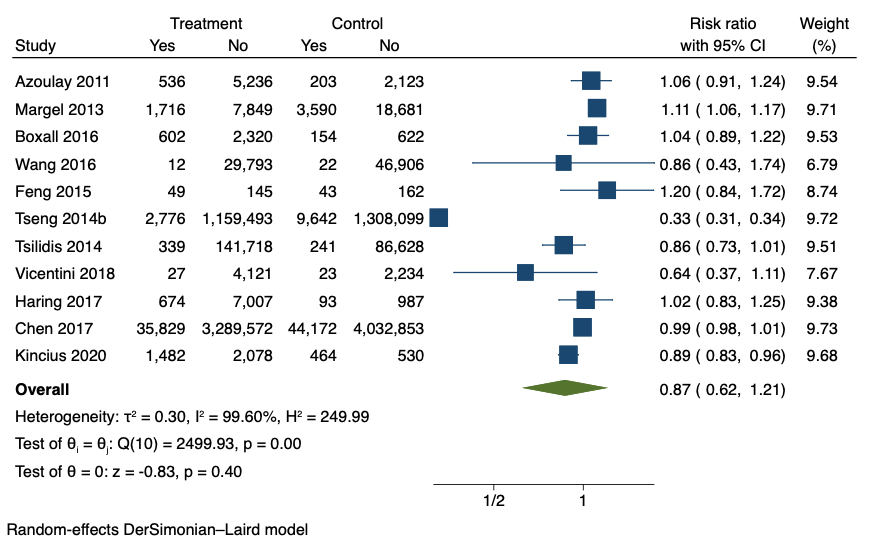
**

**Supplementary Figure 18. Pooled analysis of the association between insulin secretagogues and prostate cancer risk**

**
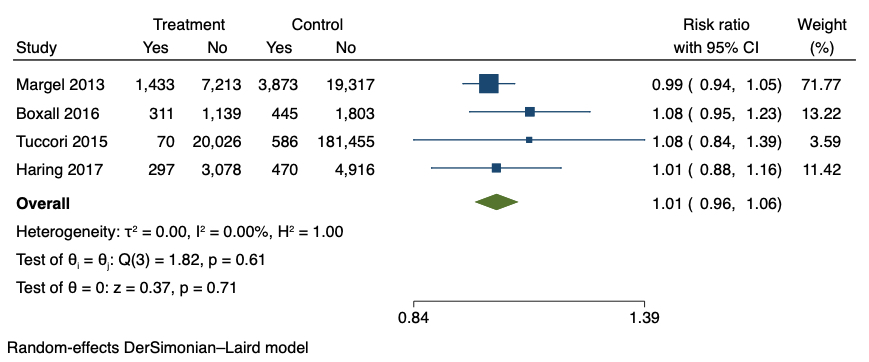
**

**Supplementary Figure 19. Pooled analysis of the association between thiazolidinediones and prostate cancer risk**

**
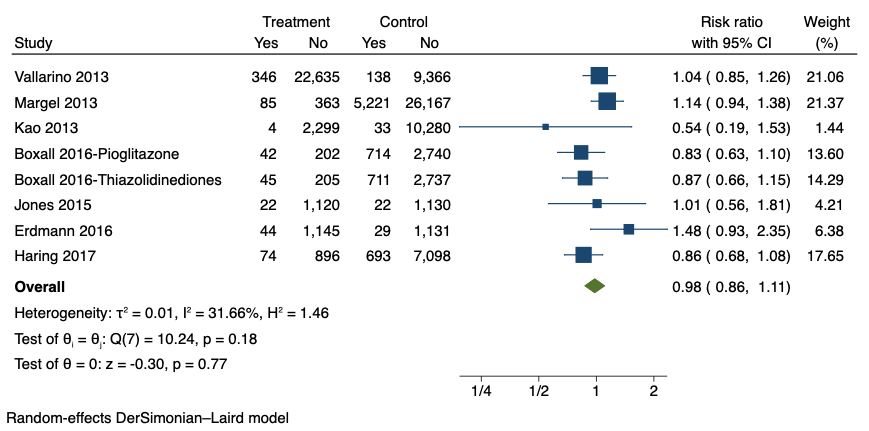
**

**Supplementary Figure 20. Pooled analysis of the association between insulins and prostate cancer risk**

**
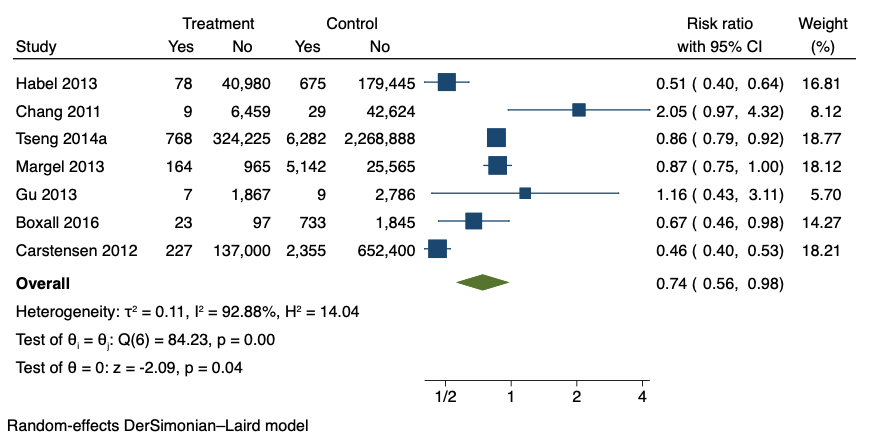
**

**Supplementary Figure 21. Pooled analysis of the association between biguanides and liver cancer risk**

**
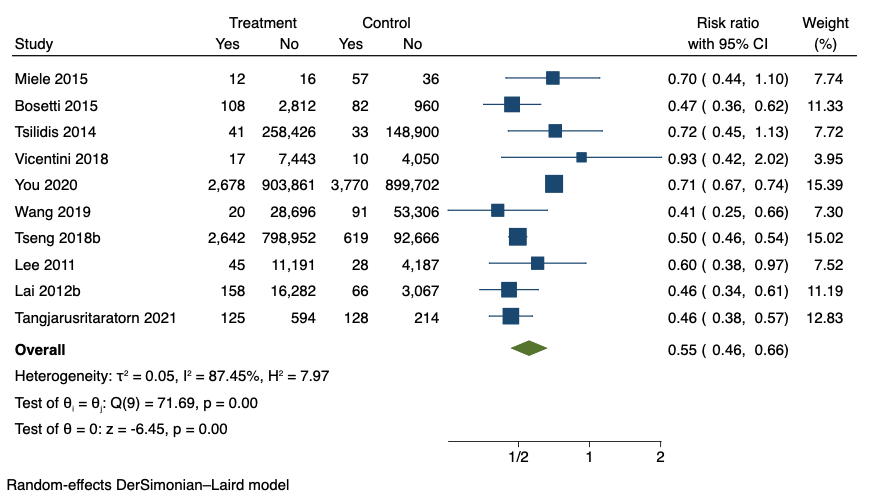
**

**Supplementary Figure 22. Pooled analysis of the association between incretin-based medicines and liver cancer risk**

**
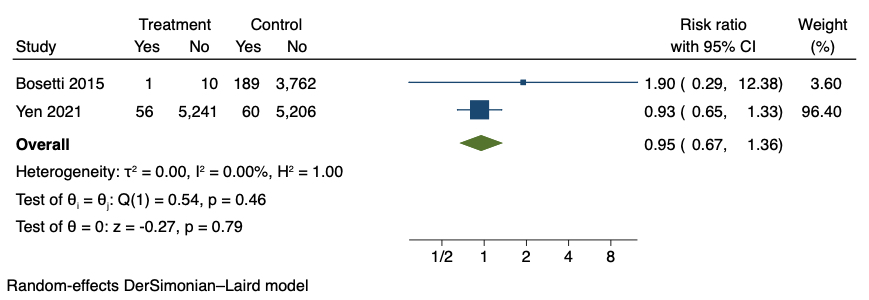
**

**Supplementary Figure 23. Pooled analysis of the association between alpha-glucosidase inhibitors and liver cancer risk**

**
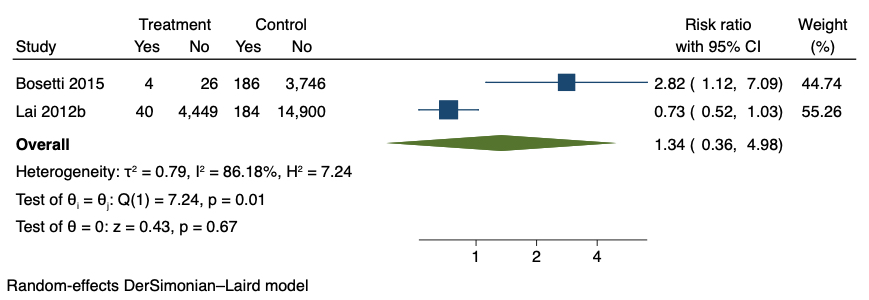
**

**Supplementary Figure 24. Pooled analysis of the association between insulin secretagogues and liver cancer risk**

**
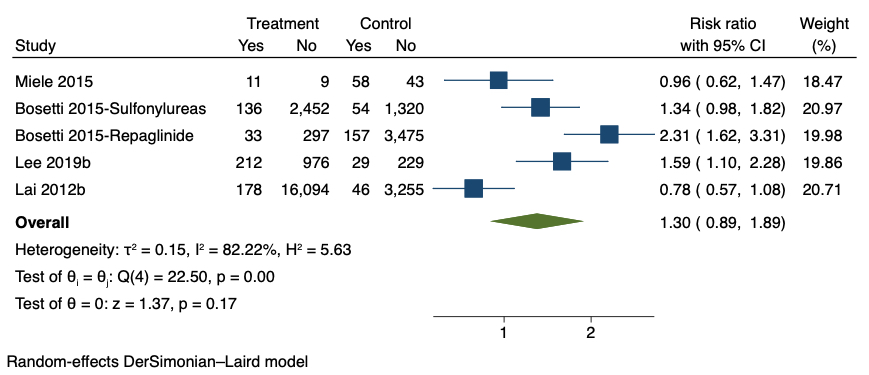
**

**Supplementary Figure 25. Pooled analysis of the association between thiazolidinediones and liver cancer risk**

**
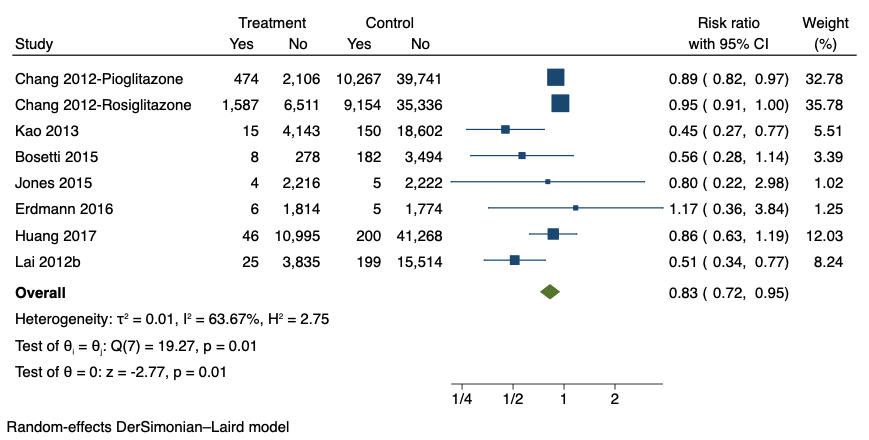
**

**Supplementary Figure 26. Pooled analysis of the association between insulins and liver cancer risk**

**
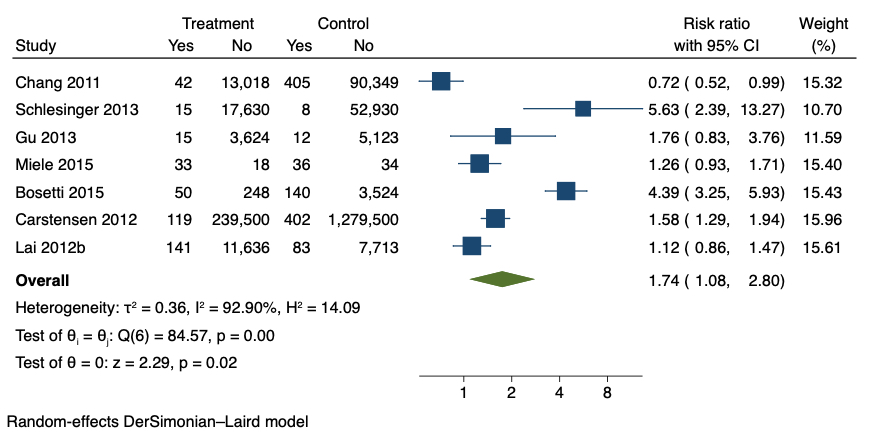
**

**Supplementary Figure 27. Pooled analysis of the association between biguanides and pancreatic cancer risk**

**
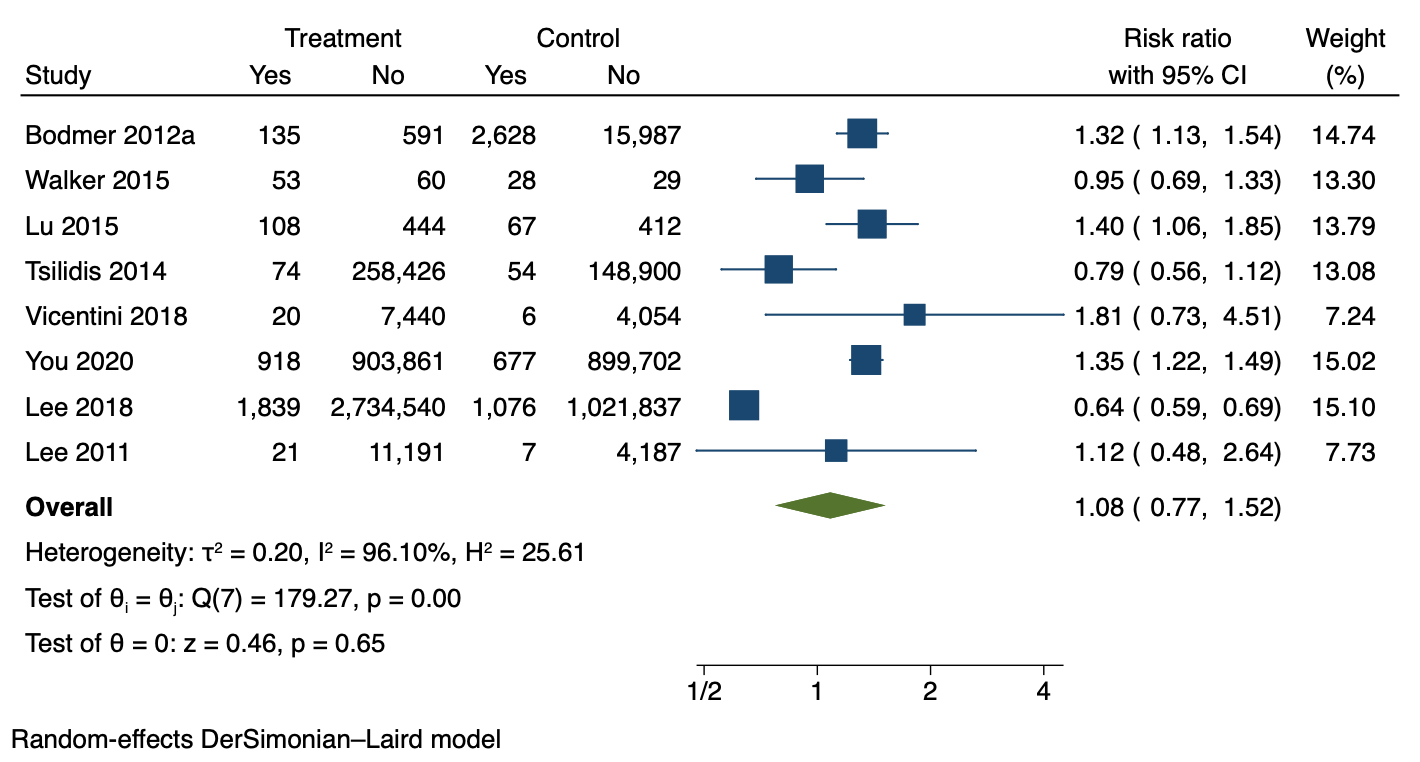
**

**Supplementary Figure 28. Pooled analysis of the association between incretin-based medicines and pancreatic cancer risk**

**
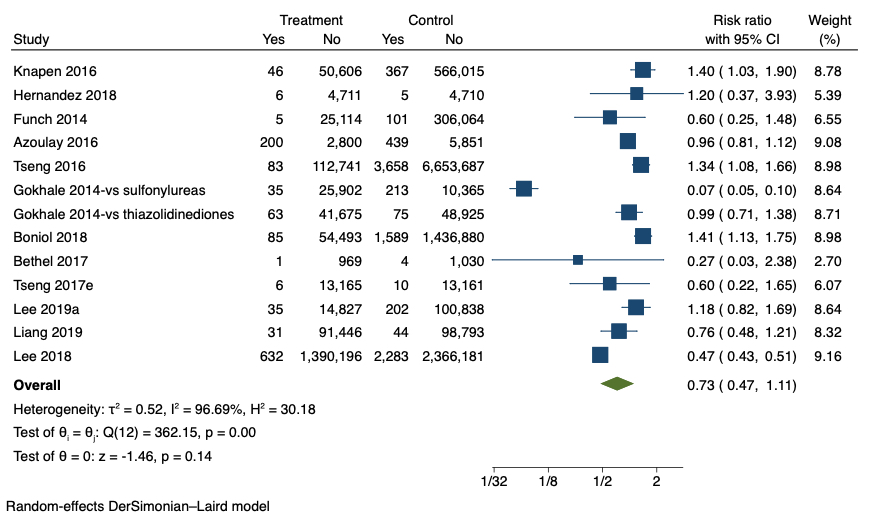
**

**Supplementary Figure 29. Pooled analysis of the association between insulin secretagogues and pancreatic cancer risk**

**
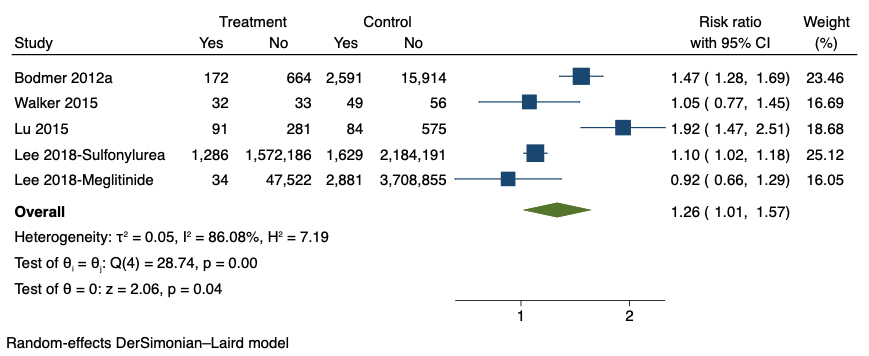
**

**Supplementary Figure 30. Pooled analysis of the association between thiazolidinediones and pancreatic cancer risk**

**
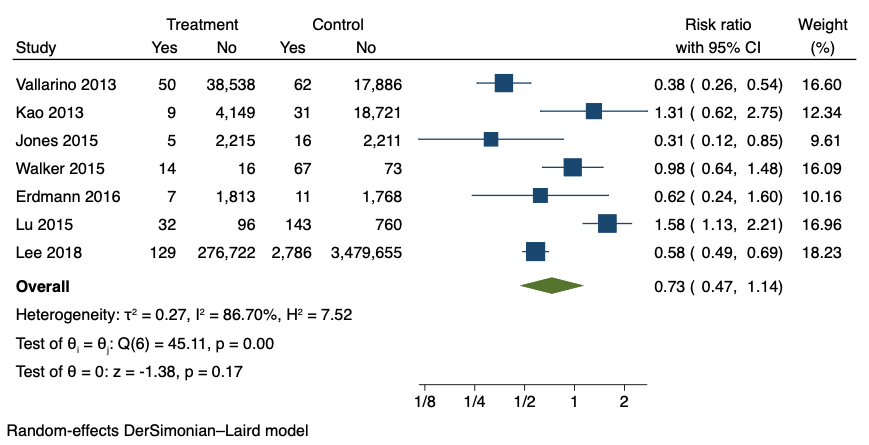
**

**Supplementary Figure 31. Pooled analysis of the association between insulins and pancreatic cancer risk**

**
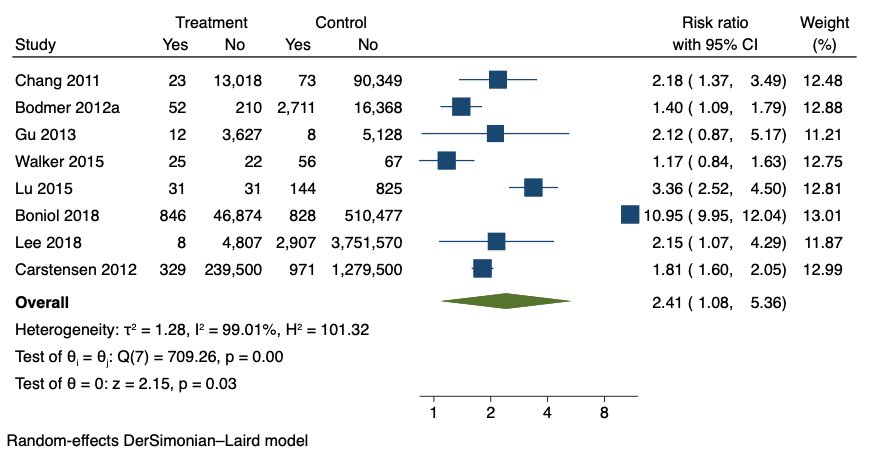
**
