# Supplementary material for: Diabetes medications and cancer risk associations: a systematic review and meta-analysis of evidence over the past 10 years
Source: Sci Rep. 2023 Jul 22;13:11844. doi: 10.1038/s41598-023-38431-z (PMC10363143; doi:10.1038/s41598-023-38431-z)
Supplement: Supplementary file 4 — Supplementary Information 4. [file 41598_2023_38431_MOESM4_ESM.docx]

**Diabetes medications and cancer risk associations: a systematic review and meta-analysis of evidence over the past 10 years**

**Author Details**

* Yixian Chen: School of Population and Public Health, University of British Columbia, Vancouver, Canada. ychen153@student.ubc.ca

Fidela Mushashi: BC Cancer, Vancouver, Canada. fidela.mushashi@bccancer.bc.ca

Surim Son: Department of Epidemiology and Biostatistics, Schulich School of Medicine & Dentistry, Western University, London, Ontario, Canada. sson8@uwo.ca

Parveen Bhatti: Cancer Control Research, BC Cancer and School of Population and Public Health, University of British Columbia, Vancouver, Canada. pbhatti@bccrc.ca

Trevor Dummer: School of Population and Public Health, University of British Columbia, Vancouver, Canada. trevor.dummer@ubc.ca

Rachel A. Murphy: Cancer Control Research, BC Cancer and School of Population and Public Health, University of British Columbia, Vancouver, Canada. rachel.murphy@ubc.ca

**Corresponding Author**

Yixian Chen: School of Population and Public Health, University of British Columbia, Vancouver, Canada. ychen153@student.ubc.ca

Mailing address: Rm 167-2206 East Mall, Vancouver, BC V6T 1Z3, Canada

**SUPPLEMENTARY FILE 4**

**Database 1: Search strategy for MEDLINE (Ovid)**

| **#** | **Query** |
| --- | --- |
| 1 | exp Hypoglycemic Agents/ |
| 2 | exp Diabetes Mellitus, Type 2/dt [drug therapy] |
| 3 | exp Diabetes Mellitus/dt [drug therapy] |
| 4 | exp Hyperglycemia/dt [drug therapy] |
| 5 | or/1-4 |
| 6 | ((diabet* or antidiabet* or anti-diabet* or hypoglycemic or antihyperglycem* or anti-hyperglycem*) adj5 (drug* or medication* or medicine* or treatment* or intervention* or therap* or medicament* or pharmaceut* or regimen)).mp. |
| 7 | ((insulin resistan* or pre-diabet* or prediabet* or pre diabet*) adj5 (drug* or medication* or medicine* or treatment* or intervention* or therap* or medicament* or pharmaceut* or regimen)).mp. |
| 8 | (metformin or biguanide or AGI or AGIs or Alpha-glucosidase inhibitor* or dipeptidyl-peptidase 4 inhibitor* or DPP 4 inhibitor* or Dipeptidyl peptidase-4 inhibitor* or glucagon-like peptide-1 or incretin* or Sitagliptin or glucagon-like peptide-1 or chemoprevention or alpha-glucosidase inhibitor or Gastric inhibitory polypeptide* or gastric inhibitory peptide*or glucose-dependent insulinotropic polypeptide* or sodium-glucose cotransporter 2 inhibitor* or SGLT2 inhibitor* or Canagliflozin or Acarbose or Miglitol or Voglibose or Exenatide or Liraglutide or Glipizide or Glybenclamide or Glibenclamide or Pioglitazone or Glitazones or Insulin glargine or insulin or Alogliptin or Linagliptin or Saxagliptin or Sitagliptin or Albiglutide or Dulaglutide or Exenatide or Liraglutide or Lixisenatide or Semaglutide or Gliptin* or Sodium Glucose Transporter 2 Inhibitor* or Sodium-Glucose Transporter 2 Inhibitor* or SGLT-2 Inhibitor* or SGLT 2 Inhibitor* or Gliflozin* or DPP-4 Inhibitor* or DPP 4 Inhibitor* or DPP-IV Inhibitor* or DPP IV Inhibitor* or Dipeptidyl Peptidase 4 Inhibitor* or Dipeptidyl-Peptidase IV Inhibitor* or Dipeptidyl Peptidase IV Inhibitor* or thiazolidinedione*).mp. |
| 9 | or/6-8 |
| 10 | 5 or 9 |
| 11 | exp Neoplasms/ |
| 12 | exp Carcinoma/ |
| 13 | exp Carcinoma, Hepatocellular/ |
| 14 | exp Liver Neoplasms/ |
| 15 | exp Pancreatic Neoplasms/ |
| 16 | exp Colorectal Neoplasms/ |
| 17 | exp Breast Neoplasms/ |
| 18 | exp Prostatic Neoplasms/ |
| 19 | exp lung neoplasms/ |
| 20 | or/12-19 |
| 21 | morbidity/ or incidence/ or mortality/ or "cause of death"/ or fatal outcome/ or mortality, premature/ or survival rate/ |
| 22 | 20 and 21 |
| 23 | ((neoplasm* or carcinogen* or cancer* or tumor or tumour or carcinoma or tumorigen*) adj5 (inciden* or diagnos* or occurrence or risk or mortality or death* or fatal*)).mp. |
| 24 | ((Breast or lung or prostate or colorectal or colon or rectal or rectum or liver or hepatic or hepato* or pancrea*) adj5 (cancer* or tumor* or tumour* or carcinoma or neoplasm*)).mp. |
| 25 | (inciden* or diagnos* or occurrence or risk or mortality or death* or fatal*).mp. |
| 26 | 24 and 25 |
| 27 | 22 or 23 or 26 |
| 28 | 10 and 27 |
| 29 | limit 28 to "humans only (removes records about animals)" |
| 30 | limit 29 to children |
| 31 | 29 not 30 |
| 32 | limit 31 to yr="2011 -Current" |
| 33 | (randomi?ed controlled trial or controlled clinical trial).pt. |
| 34 | (Randomi?ed or randomly or trial or groups).ab. |
| 35 | drug therapy.fs. |
| 36 | 33 or 34 or 35 |
| 37 | Epidemiologic studies/ |
| 38 | exp case control studies/ |
| 39 | exp cohort studies/ |
| 40 | Case control.tw. |
| 41 | (cohort adj (study or studies)).tw. |
| 42 | Cohort analy$.tw. |
| 43 | (Follow up adj (study or studies)).tw. |
| 44 | (observational adj (study or studies)).tw. |
| 45 | Longitudinal.tw. |
| 46 | Retrospective.tw. |
| 47 | or/37-46 |
| 48 | 36 or 47 |
| 49 | 32 and 48 |

**Database 2: Search strategy for Embase (Ovid)**

| **#** | **Query** |
| --- | --- |
| 1 | *antidiabetic agent/ae, ct, cm, th [Adverse Drug Reaction, Clinical Trial, Drug Comparison, Therapy] |
| 2 | ((diabet* or antidiabet* or anti-diabet* or hypoglycemic or antihyperglycem* or anti-hyperglycem*) adj5 (drug* or medication* or medicine* or treatment* or intervention* or therap* or medicament* or pharmaceut* or regimen)).mp. |
| 3 | (insulin resistan* adj5 (drug* or medication* or medicine* or treatment* or intervention* or therap* or medicament* or pharmaceut* or regimen)).mp. |
| 4 | (metformin or biguanide or AGI or AGIs or Alpha-glucosidase inhibitor* or dipeptidyl-peptidase 4 inhibitor* or DPP 4 inhibitor* or Dipeptidyl peptidase-4 inhibitor* or glucagon-like peptide-1 or incretin* or Sitagliptin or glucagon-like peptide-1 or chemoprevention or alpha-glucosidase inhibitor or Gastric inhibitory polypeptide* or gastric inhibitory peptide*or glucose-dependent insulinotropic polypeptide* or sodium-glucose cotransporter 2 inhibitor* or SGLT2 inhibitor* or Canagliflozin or Acarbose or Miglitol or Voglibose or Exenatide or Liraglutide or Glipizide or Glybenclamide or Glibenclamide or Pioglitazone or Glitazones or Insulin glargine or insulin or Alogliptin or Linagliptin or Saxagliptin or Sitagliptin or Albiglutide or Dulaglutide or Exenatide or Liraglutide or Lixisenatide or Semaglutide or Gliptin* or Sodium Glucose Transporter 2 Inhibitor* or Sodium-Glucose Transporter 2 Inhibitor* or SGLT-2 Inhibitor* or SGLT 2 Inhibitor* or Gliflozin* or DPP-4 Inhibitor* or DPP 4 Inhibitor* or DPP-IV Inhibitor* or DPP IV Inhibitor* or Dipeptidyl Peptidase 4 Inhibitor* or Dipeptidyl-Peptidase IV Inhibitor* or Dipeptidyl Peptidase IV Inhibitor* or thiazolidinedione*).mp. |
| 5 | or/1-4 |
| 6 | *malignant neoplasm/dt, ep, et, si [Drug Therapy, Epidemiology, Etiology, Side Effect] |
| 7 | *liver cancer/dt, ep, et, si [Drug Therapy, Epidemiology, Etiology, Side Effect] |
| 8 | *pancreas cancer/dt, ep, et, si [Drug Therapy, Epidemiology, Etiology, Side Effect] |
| 9 | *colorectal cancer/dt, ep, et, si [Drug Therapy, Epidemiology, Etiology, Side Effect] |
| 10 | *breast cancer/dt, ep, et, si [Drug Therapy, Epidemiology, Etiology, Side Effect] |
| 11 | *prostate cancer/dt, ep, et, si [Drug Therapy, Epidemiology, Etiology, Side Effect] |
| 12 | *lung cancer/dt, ep, et, si [Drug Therapy, Epidemiology, Etiology, Side Effect] |
| 13 | or/6-12 |
| 14 | morbidity/ or incidence/ or mortality/ or "cause of death"/ or fatal outcome/ or mortality, premature/ or survival rate/ |
| 15 | 13 and 14 |
| 16 | ((neoplasm* or carcinogen* or cancer* or tumor or tumour or carcinoma or tumorigen*) adj5 (inciden* or diagnos* or occurrence or risk or mortality or death* or fatal*)).mp. |
| 17 | ((Breast or lung or prostate or colorectal or colon or rectal or rectum or liver or hepatic or hepato* or pancrea*) adj5 (cancer* or tumor* or tumour* or carcinoma or neoplasm*)).mp. |
| 18 | (inciden* or diagnos* or occurrence or risk or mortality or death* or fatal*).mp. |
| 19 | 17 and 18 |
| 20 | 15 or 16 or 19 |
| 21 | 5 and 20 |
| 22 | (Randomized controlled trial/ or Controlled clinical study/ or random*.ti,ab. or randomization/ or intermethod comparison/ or placebo.ti,ab. or (compare or compared or comparison).ti. or ((evaluated or evaluate or evaluating or assessed or assess) and (compare or compared or comparing or comparison)).ab. or (open adj label).ti,ab. or ((double or single or doubly or singly) adj (blind or blinded or blindly)).ti,ab. or double blind procedure/ or parallel group*1.ti,ab. or (crossover or cross over).ti,ab. or ((assign* or match or matched or allocation) adj5 (alternate or group*1 or intervention*1 or patient*1 or subject*1 or participant*1)).ti,ab. or (assigned or allocated).ti,ab. or (controlled adj7 (study or design or trial)).ti,ab. or (volunteer or volunteers).ti,ab. or human experiment/ or trial.ti.) not (((random* adj sampl* adj7 ("cross section*" or questionnaire*1 or survey* or database*1)).ti,ab. not (comparative study/ or controlled study/ or randomi?ed controlled.ti,ab. or randomly assigned.ti,ab.)) or (Cross-sectional study/ not (randomized controlled trial/ or controlled clinical study/ or controlled study/ or randomi?ed controlled.ti,ab. or control group*1.ti,ab.)) or (((case adj control*) and random*) not randomi?ed controlled).ti,ab. or (Systematic review not (trial or study)).ti. or (nonrandom* not random*).ti,ab. or "Random field*".ti,ab. or (random cluster adj3 sampl*).ti,ab. or ((review.ab. and review.pt.) not trial.ti.) or ("we searched".ab. and (review.ti. or review.pt.)) or "update review".ab. or (databases adj4 searched).ab. or ((rat or rats or mouse or mice or swine or porcine or murine or sheep or lambs or pigs or piglets or rabbit or rabbits or cat or cats or dog or dogs or cattle or bovine or monkey or monkeys or trout or marmoset*1).ti. and animal experiment/) or (Animal experiment/ not (human experiment/ or human/))) |
| 23 | Epidemiologic studies/ or exp case control studies/ or exp cohort studies/ or Case control.tw. or (cohort adj (study or studies)).tw. or Cohort analy$.tw. or (Follow up adj (study or studies)).tw. or (observational adj (study or studies)).tw. or Longitudinal.tw. or Retrospective.tw. |
| 24 | 22 or 23 |
| 25 | 21 and 24 |
| 26 | limit 25 to "humans only (removes records about animals)" |
| 27 | limit 26 to children |
| 28 | 26 not 27 |
| 29 | limit 28 to yr="2011 -Current" |

**Database 3: Search strategy for Web of Science Core Collection**

| **#** | **Query** |
| --- | --- |
| 1 | TI=(breast or lung or prostate or colorectal or colon or rectal or rectum or liver or hepatic or hepato* or pancrea*) |
| 2 | TI=(neoplasms or carcinogen* or cancer* or tumor or tumour or carcinoma or tumorigen*) |
| 3 | 2 AND 1 |
| 4 | TI=(morbidity or mortalit* or death* or fatal* or inciden* or risk or diagnos* or occurrence) |
| 5 | 4 AND 3 |
| 6 | TS=(hypoglycemic or antihyperglycemic or anti-hyperglycemic or anti hyperglycemic or diabet* or antidiabet* or anti-diabet* or anti diabet* or insulin resistan* or prediabet* or pre-diabet* or pre diabet* ) |
| 7 | TS=(agent* or drug* or medication* or medicine* or treatment* or intervention* or therap* or medicament* or pharmaceut* or regimen) |
| 8 | 7 AND 6 |
| 9 | TS=(metformin or biguanide or AGI or AGIs or Alpha-glucosidase inhibitor* or dipeptidyl-peptidase 4 inhibitor* or DPP 4 inhibitor* or Dipeptidyl peptidase-4 inhibitor* or glucagon-like peptide-1 or incretin* or Sitagliptin or glucagon-like peptide-1 or chemoprevention or alpha-glucosidase inhibitor or Gastric inhibitory polypeptide* or gastric inhibitory peptide*or glucose-dependent insulinotropic polypeptide* or sodium-glucose cotransporter 2 inhibitor* or SGLT2 inhibitor* or Canagliflozin or Acarbose or Miglitol or Voglibose or Exenatide or Liraglutide or Glipizide or Glybenclamide or Glibenclamide or Pioglitazone or Glitazones or Insulin glargine or insulin or Alogliptin or Linagliptin or Saxagliptin or Sitagliptin or Albiglutide or Dulaglutide or Exenatide or Liraglutide or Lixisenatide or Semaglutide or Gliptin* or Sodium Glucose Transporter 2 Inhibitor* or Sodium-Glucose Transporter 2 Inhibitor* or SGLT-2 Inhibitor* or SGLT 2 Inhibitor* or Gliflozin* or DPP-4 Inhibitor* or DPP 4 Inhibitor* or DPP-IV Inhibitor* or DPP IV Inhibitor* or Dipeptidyl Peptidase 4 Inhibitor* or Dipeptidyl-Peptidase IV Inhibitor* or Dipeptidyl Peptidase IV Inhibitor* or thiazolidinedione*) |
| 10 | 9 OR 8 |
| 11 | 10 AND 5 |
| 12 | TS=(randomised OR randomized OR randomisation OR randomisation OR placebo* OR (random* AND (allocat* OR assign*) ) OR (blind* AND (single OR double OR treble OR triple) )) NOT TS=(animal or animals or pisces or fish or fishes or catfish or catfishes or sheatfish or silurus or arius or heteropneustes or clarias or gariepinus or fathead minnow or fathead minnows or pimephales or promelas or cichlidae or trout or trouts or char or chars or salvelinus or salmo or oncorhynchus or guppy or guppies or millionfish or poecilia or goldfish or goldfishes or carassius or auratus or mullet or mullets or mugil or curema or shark or sharks or cod or cods or gadus or morhua or carp or carps or cyprinus or carpio or killifish or eel or eels or anguilla or zander or sander or lucioperca or stizostedion or turbot or turbots or psetta or flatfish or flatfishes or plaice or pleuronectes or platessa or tilapia or tilapias or oreochromis or sarotherodon or common sole or dover sole or solea or zebrafish or zebrafishes or danio or rerio or seabass or dicentrarchus or labrax or morone or lamprey or lampreys or petromyzon or pumpkinseed or pumpkinseeds or lepomis or gibbosus or herring or clupea or harengus or amphibia or amphibian or amphibians or anura or salientia or frog or frogs or rana or toad or toads or bufo or xenopus or laevis or bombina or epidalea or calamita or salamander or salamanders or newt or newts or triturus or reptilia or reptile or reptiles or bearded dragon or pogona or vitticeps or iguana or iguanas or lizard or lizards or anguis fragilis or turtle or turtles or snakes or snake or aves or bird or birds or quail or quails or coturnix or bobwhite or colinus or virginianus or poultry or poultries or fowl or fowls or chicken or chickens or gallus or zebra finch or taeniopygia or guttata or canary or canaries or serinus or canaria or parakeet or parakeets or grasskeet or parrot or parrots or psittacine or psittacines or shelduck or tadorna or goose or geese or branta or leucopsis or woodlark or lullula or flycatcher or ficedula or hypoleuca or dove or doves or geopelia or cuneata or duck or ducks or greylag or graylag or anser or harrier or circus pygargus or red knot or great knot or calidris or canutus or godwit or limosa or lapponica or meleagris or gallopavo or jackdaw or corvus or monedula or ruff or philomachus or pugnax or lapwing or peewit or plover or vanellus or swan or cygnus or columbianus or bewickii or gull or chroicocephalus or ridibundus or albifrons or great tit or parus or aythya or fuligula or streptopelia or risoria or spoonbill or platalea or leucorodia or blackbird or turdus or merula or blue tit or cyanistes or pigeon or pigeons or columba or pintail or anas or starling or sturnus or owl or athene noctua or pochard or ferina or cockatiel or nymphicus or hollandicus or skylark or alauda or tern or sterna or teal or crecca or oystercatcher or haematopus or ostralegus or shrew or shrews or sorex or araneus or crocidura or russula or european mole or talpa or chiroptera or bat or bats or eptesicus or serotinus or myotis or dasycneme or daubentonii or pipistrelle or pipistrellus or cat or cats or felis or catus or feline or dog or dogs or canis or canine or canines or otter or otters or lutra or badger or badgers or meles or fitchew or fitch or foumart or foulmart or ferrets or ferret or polecat or polecats or mustela or putorius or weasel or weasels or fox or foxes or vulpes or common seal or phoca or vitulina or grey seal or halichoerus or horse or horses or equus or equine or equidae or donkey or donkeys or mule or mules or pig or pigs or swine or swines or hog or hogs or boar or boars or porcine or piglet or piglets or sus or scrofa or llama or llamas or lama or glama or deer or deers or cervus or elaphus or cow or cows or bos taurus or bos indicus or bovine or bull or bulls or cattle or bison or bisons or sheep or sheeps or ovis aries or ovine or lamb or lambs or mouflon or mouflons or goat or goats or capra or caprine or chamois or rupicapra or leporidae or lagomorpha or lagomorph or rabbit or rabbits or oryctolagus or cuniculus or laprine or hares or lepus or rodentia or rodent or rodents or murinae or mouse or mice or mus or musculus or murine or woodmouse or apodemus or rat or rats or rattus or norvegicus or guinea pig or guinea pigs or cavia or porcellus or hamster or hamsters or mesocricetus or cricetulus or cricetus or gerbil or gerbils or jird or jirds or meriones or unguiculatus or jerboa or jerboas or jaculus or chinchilla or chinchillas or beaver or beavers or castor fiber or castor canadensis or sciuridae or squirrel or squirrels or sciurus or chipmunk or chipmunks or marmot or marmots or marmota or suslik or susliks or spermophilus or cynomys or cottonrat or cottonrats or sigmodon or vole or voles or microtus or myodes or glareolus or primate or primates or prosimian or prosimians or lemur or lemurs or lemuridae or loris or bush baby or bush babies or bushbaby or bushbabies or galago or galagos or anthropoidea or anthropoids or simian or simians or monkey or monkeys or marmoset or marmosets or callithrix or cebuella or tamarin or tamarins or saguinus or leontopithecus or squirrel monkey or squirrel monkeys or saimiri or night monkey or night monkeys or owl monkey or owl monkeys or douroucoulis or aotus or spider monkey or spider monkeys or ateles or baboon or baboons or papio or rhesus monkey or macaque or macaca or mulatta or cynomolgus or fascicularis or green monkey or green monkeys or chlorocebus or vervet or vervets or pygerythrus or hominoidea or ape or apes or hylobatidae or gibbon or gibbons or siamang or siamangs or nomascus or symphalangus or hominidae or orangutan or orangutans or pongo or chimpanzee or chimpanzees or pan troglodytes or bonobo or bonobos or pan paniscus or gorilla or gorillas or troglodytes) |
| 13 | TS=(observational stud* or epidemiologic stud* or case control stud* or case-control stud* or case control or cohort stud* or cohort analysis or cohort analyses or follow up stud* or follow-up stud* or longitudinal) |
| 14 | 13 OR 12 |
| 15 | 14 AND 11 |
| 16 | 14 AND 11  Refined by: [excluding] PUBLICATION YEARS: ( 2005 OR 1997 OR 2004 OR 1996 OR 2003 OR 1995 OR 2010 OR 2002 OR 1994 OR 2009 OR 2001 OR 1993 OR 2008 OR 2000 OR 1992 OR 2007 OR 1999 OR 1991 OR 2006 OR 1998 ) |

**Database 4: Search strategy for Cochrane CENTRAL (Ovid)**

| **#** | **Query** |
| --- | --- |
| 1 | exp hypoglycemic agents/ |
| 2 | ((diabet* or antidiabet* or anti-diabet* or hypoglycemic or antihyperglycem* or anti-hyperglycem*) adj5 (drug* or medication* or medicine* or treatment* or intervention* or therap* or medicament* or pharmaceut* or regimen)).mp. |
| 3 | ((insulin resistan* or pre-diabet* or prediabet* or pre diabet*) adj5 (drug* or medication* or medicine* or treatment* or intervention* or therap* or medicament* or pharmaceut* or regimen)).mp. |
| 4 | (metformin or biguanide or AGI or AGIs or Alpha-glucosidase inhibitor* or dipeptidyl-peptidase 4 inhibitor* or DPP 4 inhibitor* or Dipeptidyl peptidase-4 inhibitor* or glucagon-like peptide-1 or incretin* or Sitagliptin or glucagon-like peptide-1 or chemoprevention or alpha-glucosidase inhibitor or Gastric inhibitory polypeptide* or gastric inhibitory peptide*or glucose-dependent insulinotropic polypeptide* or sodium-glucose cotransporter 2 inhibitor* or SGLT2 inhibitor* or Canagliflozin or Acarbose or Miglitol or Voglibose or Exenatide or Liraglutide or Glipizide or Glybenclamide or Glibenclamide or Pioglitazone or Glitazones or Insulin glargine or insulin or Alogliptin or Linagliptin or Saxagliptin or Sitagliptin or Albiglutide or Dulaglutide or Exenatide or Liraglutide or Lixisenatide or Semaglutide or Gliptin* or Sodium Glucose Transporter 2 Inhibitor* or Sodium-Glucose Transporter 2 Inhibitor* or SGLT-2 Inhibitor* or SGLT 2 Inhibitor* or Gliflozin* or DPP-4 Inhibitor* or DPP 4 Inhibitor* or DPP-IV Inhibitor* or DPP IV Inhibitor* or Dipeptidyl Peptidase 4 Inhibitor* or Dipeptidyl-Peptidase IV Inhibitor* or Dipeptidyl Peptidase IV Inhibitor* or thiazolidinedione*).mp. |
| 5 | or/1-4 |
| 6 | Neoplasm*/ or breast neoplasms/ or liver neoplasms/ or pancreatic neoplasms/ or lung neoplasms/ or prostatic neoplasms/ or colorectal neoplasms/ or colonic neoplasms/ or rectal neoplasms/ |
| 7 | morbidity/ or incidence/ or mortality/ or "cause of death"/ or fatal outcome/ or mortality, premature/ or survival rate/ |
| 8 | 6 and 7 |
| 9 | ((neoplasms or carcinogen* or cancer* or tumor or tumour or carcinoma or tumorigen*) adj5 (inciden* or diagnos* or occurrence or risk or mortality or death* or fatal*)).mp. |
| 10 | ((Breast or lung or prostate or colorectal or colon or rectal or rectum or liver or hepatic or hepato* or pancrea*) adj5 (cancer* or tumor* or tumour* or carcinoma or neoplasm*)).mp. |
| 11 | (inciden* or diagnos* or occurrence or risk or mortality or death* or fatal*).mp. |
| 12 | 10 and 11 |
| 13 | 8 or 9 or 12 |
| 14 | (randomi?ed controlled trial or controlled clinical trial).pt. |
| 15 | (Randomi?ed or randomly or trial or groups).ab. |
| 16 | 14 or 15 |
| 17 | Epidemiologic studies/ |
| 18 | exp case control studies/ |
| 19 | exp cohort studies/ |
| 20 | Case control.tw. |
| 21 | (cohort adj (study or studies)).tw. |
| 22 | Cohort analy$.tw. |
| 23 | (Follow up adj (study or studies)).tw. |
| 24 | (observational adj (study or studies)).tw. |
| 25 | Longitudinal.tw. |
| 26 | Retrospective.tw. |
| 27 | or/17-26 |
| 28 | 16 or 27 |
| 29 | 5 and 13 and 28 |
| 30 | limit 29 to yr="2011 -Current" |
| 31 | 30 not 27 |
